# Effects of phase transition and thermodynamics on the dynamics of cavitation bubbles collapsing near a solid boundary and the associated 3D flow

**DOI:** 10.1016/j.ultsonch.2026.107894

**Published:** 2026-05-22

**Authors:** Udo Lantermann, Gohar Moloudi, Mazyar Dawoodian, Hemant J. Sagar, Ould el Moctar

**Affiliations:** a Institute of Sustainable and Autonomous Maritime Systems, University of Duisburg–Essen, Duisburg, Germany; b Department of Hydro and Renewable Energy, IIT Roorkee, Roorkee, India; c Chair of Autonomous and Energy Efficient Maritime Technologies, Technische Universität Berlin, Berlin, Germany

**Keywords:** Phase transition, Cavitation, Bubble collapse, Thermal effects, 3D flow

## Abstract

This paper documents investigated phase transition as well as thermal and water salinity effects on the dynamics of three-dimensional cavitation bubbles collapsing near solid boundaries by employing mathematical models that account for phase transition, compressibility, thermodynamic, and three-dimensional effects. We performed systematic three-dimensional simulations of the two-phase flow by solving the coupled conservation equations of mass, momentum, and energy as well as a transport equation for the volume fraction using a mass transfer model. We conducted extensive verification and validation studies. Results showed that phase transition affected the bubble dynamics substantially, albeit mainly after the bubble’s first collapse. After the initial bubble collapse, the vapor phase heated up to about 460 K. The simulations captured asymmetric jets and toroidal collapse structures, while the experiments on salinity revealed that increased salt concentration weakened bubble rebound and left persistent vapor remnants. The induced pressures, temperatures, and velocities from bubble collapse are presented and discussed.

## Highlights

• Three-dimensional simulations reveal thermodynamic and phase-change effects in near-wall cavitation bubble collapse.
• Phase transition strongly influence collapse pressure, temperature, and jet dynamics.
• Salinity has a minor influence on bubble dynamics and slightly affects collapse behavior near solid boundaries.

## Graphical abstract

**Figure:**
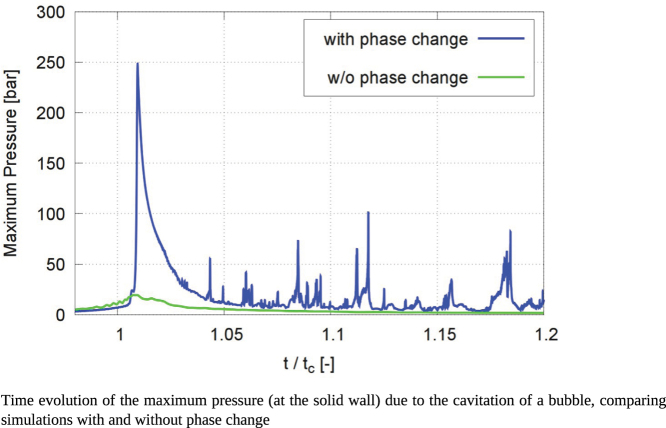

## 1 Introduction

Cavitation is a dynamic phase transition phenomenon that occurs in liquids when the local pressure drops below the saturation pressure of the liquid, leading to the formation and collapse of vapor bubbles. It is essential in many engineering applications, such as marine propellers, heating systems, water treatment, and surface cleaning systems [1], [2], [3], [4], [5].

Since the early stages of cavitation research, experimental [6], [7], [8], [9] and numerical approaches [10], [11], [12], [13], [14] have been continuously developed and applied to capture bubble dynamics phenomena in different environments and near various boundaries and geometries. When cavitation bubbles collapse near a rigid surface, high-speed microjets are generated towards the wall, reaching velocities of several hundred meters per second during the first collapse [15], [16], [17]. Microjets deform the bubble into a toroidal shape and generate shock waves upon impact with the surface. As the toroidal bubble collapses close to the boundary, a secondary microjet and shock waves may form due to a repetitive growth and collapse near the surface. Following the first collapse, this subsequent collapse may further damage the surface following the first collapse [18], [19], [20], [21]. Lechner et al. [22], who numerically demonstrated that the collapse of a bubble near a solid wall and a cylindrical pillar may generate fast jets of about 1000 m/s. The authors showed that viscosity and geometry of the nearby boundary have a strong effect on jet dynamics.

In reality, a cavitation bubble typically contains both vapor and non-condensable gas [15], [23]. Vapor remains trapped near the center of the bubble, while the non-condensable gas at the interface diffuses slowly, especially during the bubble’s initial oscillations. The dynamics of bubbles, primarily filled with vapor, may be more complex, and the bubble’s radius may be significantly smaller during collapse, resulting in high-pressure peaks from shock waves emission. Consequently, vapor bubbles tend to collapse more violently than those containing non-condensable gas mixtures. One of the main features of bubble collapse is shock wave emission, which makes it essential to account for compressibility. Indeed, compressibility plays a significant role throughout the bubble’s entire oscillation.

Several numerical studies simplified the problem by modeling the bubble as gas-filled and the surrounding liquid as incompressible [23], [24], [25]. Such assumptions are valid for low-speed flows far below the speed of sound. These studies neglect shock waves and rapid pressure variations, leading to an underestimation of collapse intensity. Therefore, such models are inadequate to accurately capture cavitation erosion mechanisms. However, accounting for compressibility leads to higher predicted jet velocities and pressure wave magnitudes. While continuum models provide valuable insights into cavitation at microscale, they cannot resolve the molecular-level processes. Rezaee et al. [26] presented a coarse-grained molecular dynamics approach for laser-induced nanobubbles, capturing nucleation, collapse, phase transition, and interphase dynamics.

The dynamics of bubbles filled with a condensable gas, such as vapor, is highly dependent on the condensation and vaporization rate, particularly near solid surfaces [27]. During the bubble’s first collapse, pressures may reach several thousand bars [28], causing condensation of almost the entire vapor phase. At this point, the bubble primarily contains non-condensable gas. Microjet-induced pressure waves may locally reduce the pressure below saturation pressure, leading to vaporization of the surrounding liquid.

Heat transfer processes such as boiling and condensation significantly influence the dynamics of vapor bubbles in thermal fluids. The thermophysical properties of the vapor phase are highly sensitive to temperature and phase transition [29], [30]. Molecular Dynamics (MD) simulations and high-resolution numerical models have revealed that bubble collapse near a solid boundary may generate extremely high temperatures, often reaching several thousand K [31], [32]. These temperature spikes occur over very small spatial and temporal scales, raising the question of how much they affect bubble dynamics and contribute to material damage. Benjamin and Ellis [33] estimated a maximum gas temperature of about 8800 K at the center of the bubble. By accounting for heat transfer in their model, Fujikawa and Akamatsu [34] reported a peak temperature of 6700 K and a pressure of 848 bar. These values were followed by an immediate drop to ambient conditions. Okhotsimskii [4] analyzed the thermally driven bubble collapse, and they predicted complete condensation when the Jakob number falls below 0.1. Experimental research on cavitation erosion by Haosheng et al. [16] discovered that internal temperatures during collapse may reach up to 10,000 °C. However, the thin water layer between the collapsing bubble and the solid wall diminishes the thermal effects by limiting and disturbing heat transfer. A heat-affected zone, indicating exposure to temperatures above 300 °C, was observed around erosion pits. Dular and Coutier-Delgosha [18] investigated the thermodynamic behavior of oscillating microbubbles generated by a tube arrest technique, which avoids external thermal input from lasers or sparks. They showed that both gas compression and phase transition influence bubble volume. When the phase transition time exceeds the bubble growth time, evaporation does not have enough time to occur, which is the case during the expansion phase. Inaddition, they found that the temperature decreases about 3 K during the expansion phase and increases up to 4 K during the contraction phase.

Goncalvès [19] numerically studied cavitation in freon R-114 using a modified barotropic and stiffened gas equation of state. They showed that downstream heating during collapse dominates over cooling. Quinto et al. [29] by quantifying temperature fields of laser-induced bubbles using fluorescence thermometry, observed localized heating of up to 13 °C during short lifespans. Hamdi et al. [30] directly measured the thermal field inside and around bubbles using a cold-wire thermometer under tube arrest conditions.

Ye et al. [35] simulated bubble oscillation under multifrequency ultrasound conditions using a van der Waals equation of state. They observed that while minimum pressure and temperature decreased, maximum values increased under dual-frequency excitation. Suslick et al. [32] used spectroscopic methods to analyze single and multiple bubble cavitation. They showed that bubble temperature depends on the dissolved gas and the thermal conductivity of the vapor pressure. Zhang et al. [36] investigated energy conversion in collapsing single and clustered bubbles. They found that thermal and acoustic damping reduce conversion efficiency, particularly when asphericity delays collapse Yang et al. [12] by using a thermal Multiple Relaxation Time Lattice Boltzmann Method (LBM-MRT) approach and assuming adiabatic collapse, found localized hot spots caused by pressure buildup and insufficient vapor cooling near rigid boundaries.

Peng et al. [37] simulated bubble collapse near rigid boundaries using a thermal LBM-MRT method and the Carnahan-Starling equation of state. They showed that, beyond a stand-off distance of γ=2.2, the boundary’s influence decreases. Yu et al. [38] analyzed the jet-induced heat transfer between the bubble and the surrounding liquid. They revealed the formation of hot zones near the wall and cooler regions at the bubble center during rebound. Liu and Peng [39] studied temperature distributions during collapse near flat and curved walls using the LBM-MRT method. They observed that collapse far from the wall leads to higher peak temperatures. Qin and Alehossein [40] extended the Rayleigh–Plesset model to include conduction, convection, and radiation, finding that larger initial radius (e.g., 2 mm) lead to collapse temperatures exceeding 20,000 K, with radiation dominating heat transfer in large bubbles and conduction in smaller ones.

Duan et al. [41] investigated acoustic emissions by modeling a compressible, viscous two-phase flow using the Schnerr-Sauer cavitation model. They showed stronger jets are generated at smaller bubble to wall distances. Shan et al. [42] applied a thermal Lattice Boltzmann Method (LBM) with the Peng-Robinson equation of state to map internal temperature zones, linking them to jet velocity and initial parameters. Yin et al. [43] examined bubble pairs with Navier–Stokes equations and the volume of fluid (VoF) method, showing reduced peak temperatures due to nearby bubble interactions that are similar to wall effects. Furthermore, they classified the rebound behavior based on the stand-off parameter γ. Phan et al. [27] used a compressible mixture model and showed that low ambient temperatures leads to inertia-driven collapses, while high temperatures enhanced thermal effects.

Peng et al. [44] modeled oscillating vapor–gas, argon, bubbles with the Gilmore equation and the Hertz–Knudsen–Langmuir mass transfer model and found that below 35 °C the collapse temperature decreases with rising liquid temperature. Hegedűs et al. [45] included thermal damping in the Rayleigh framework, showing increased heat transfer at higher ambient temperatures. In sonoluminescence research, lower water temperature led to stronger light emission and higher bubble temperatures due to reduced trapped vapor [46], [47]. Liu et al. [48] experimentally demonstrated that water near 50 °C increases the impact pressure during collapse near walls due to modified thermophysical properties. Their findings consistently showed that ambient temperature and fluid properties control collapse strength, energy dissipation, and rebound dynamics. Zhang et al. [49] proposed a theoretical model for bubble dynamics that includes phase transition, migration, and boundary effects. Using a modified Hertz–Knudsen–Langmuir model and the van der Waals gas law, they simulated vapor–gas bubbles over varying Mach numbers and vapor contents. Their results showed that higher vapor fractions and Mach numbers increase energy loss and intensify collapse, generating stronger pressure peaks and acoustic emissions. Their model was validated experimentally using laser, spark, and explosion-generated bubbles in various environments. Their simulations reproduced asymmetric rebounds and bubble migration near walls, underscoring the role of compressibility and phase transition under confined conditions. This agreed with our current study, which fully captured collapse-rebound cycles near solid boundaries using a validated mass transfer model.

While bubble dynamics is inherently three-dimensional, capturing pressure waves and microjets from collapse requires fine resolutions. Early studies often simplified the problem using 1D or 2D models to reduce computational costs. For instance, 2D Rayleigh-type models were used because fully 2D hydrodynamic simulations were prohibitively expensive. The boundary element method (BEM) exploits Green’s functions to reduce the 3D Laplace equation to a 2D integral, which lowers costs and allows interface tracking. Some studies assumed spherical symmetry and simulated a sector of a bubble that exceeds 5 deg. They were able to capture some key aspects of bubble behavior. Although 2D and axisymmetric models reduce costs, they sacrifice physical realism. All 3D models, although expensive, are essential for reproducing shock waves, asymmetric jets, and thermally driven phenomena.

Our numerical study analyzed the collapse of a cavitation bubble in a fully three-dimensional domain, with particular focus on the roles of phase change, compressibility, and thermal effects. We combined a series of high-resolution simulations with experiments to examine how these factors influence bubble dynamics and the flow field surrounding the collapsing bubbles. We studied the effects of salinity experimentally. The structure of the paper is as follows. Section 2 presents the numerical model. Section 3 describes the experimental method. Sections 4 and 5 outline the simulation setup and, the verification and validation studies. Section 6 presents the main results, including four subsections on three dimensional flow features, phase transition, thermal effects, and salinity. Section 7 provides the conclusions.

## 2 Numerical method

We investigated the dynamics of a single cavitation bubble near a solid wall using a compressible two-phase model. The model employs a homogeneous mixture approach for the liquid and vapor phases. The governing equations consist of the conservation laws for mass, momentum, and energy, along with a transport equation for the liquid volume fraction. The compressible continuity equation describes the conservation of mass:

(1) $$\frac{\partial \rho }{\partial t}+\nabla \cdot \left(\rho u\right)=0,$$

where ρ is the density of the mixture and u is the velocity field. The momentum equation reads as follows:

(2) $$\frac{\partial \rho u}{\partial t}+\nabla \cdot \left(\rho uu\right)=-\nabla p+\nabla \cdot \left[\mu \left(\nabla u+{\left(\nabla u\right)}^{T}-\frac{2}{3}\left(\nabla \cdot u\right)I\right)\right]+\rho g+\sigma \kappa \nabla \alpha_{l}$$

where p is the pressure field, μ is the mixture viscosity, and I is the identity matrix. Symbol g is the vector of gravitational acceleration. The last term on the right-hand side describes the surface tension, σ, between liquid and vapor phases with the curvature κ of the interface. We calculated the surface tension force by the continuum surface model of Brackbill et al. [50], where the curvature of the interface is defined as

(3) $$\kappa =-\nabla \cdot \frac{\nabla \alpha_{l}}{\left|\nabla \alpha_{l}\right|}.$$

To describe the liquid and vapor phases, the volume fractions must satisfy the following relationship:

(4) $$\alpha_{l}+\alpha_{v}=1$$

The properties of the mixture are obtained as follows:

(5) $$\rho =\alpha_{l}\rho_{l}+\alpha_{v}\rho_{v}$$

(6) $$\mu =\alpha_{l}\mu_{l}+\alpha_{v}\mu_{v}$$

where indices l and v denote the liquid and vapor phases, respectively. To track the interface between phases and to handle the evolution of the bubble shape, the Volume-of-Fluid (VOF) method is employed. The transport equation for the liquid volume fraction $\alpha_{l}$ reads as follows:

(7) $$\frac{\partial \alpha_{l}}{\partial t}+\nabla \cdot \left(\alpha_{l}u\right)-\alpha_{l}\left(\nabla \cdot u\right)+\nabla \cdot \left[\alpha_{l}\left(1-\alpha_{l}\right)u_{r}\right]=$$

$$\alpha_{l}\left(1-\alpha_{l}\right)\left(\frac{1}{\rho_{v}}\frac{d\rho_{v}}{dt}-\frac{1}{\rho_{l}}\frac{d\rho_{l}}{dt}\right)+\left({\overset{˙}{m}}_{c}+{\overset{˙}{m}}_{v}\right)\left(\frac{1}{\rho_{l}}-\alpha_{l}\left(\frac{1}{\rho_{l}}-\frac{1}{\rho_{v}}\right)\right)$$

where ${\overset{˙}{m}}_{c}$ and ${\overset{˙}{m}}_{v}$ are the mass transfer rates per unit volume due to condensation and vaporization, respectively. The term $u_{r}=c_{\alpha }\left|u\right|\frac{\nabla \alpha_{l}}{\left|\nabla \alpha_{l}\right|}$ denotes the relative velocity between the liquid and vapor phases, where $c_{\alpha }$ is a coefficient that controls the artificial compression at the interface.

There are several models for phase transition. All these models have in common that they contain an empirical estimate. One group of mass transfer models is based on the Hertz–Knudsen model, Hertz, Knudsen [51], [52]. The Hertz–Knudsen equation originates from kinetic theory of gases and quantifies the net mass flux due to phase transition (evaporation or condensation) at a liquid–vapor interface. In its general form, the net mass flux per unit interfacial area is given by

(8) $$\overset{˙}{m}=\alpha \sqrt{\frac{M}{2\pi RT}}\left(p_{v}-p_{\mathrm{sat}}\left(T\right)\right),$$

where $\overset{˙}{m}$ is the interfacial mass flux, α is the accommodation coefficient, M is the molar mass of the vapor, R is the universal gas constant, T is the interface temperature, $p_{v}$ is the local vapor pressure, and $p_{\mathrm{sat}}$ is the saturation vapor pressure at temperature T. It estimates how much mass crosses the interface per unit area per unit time, driven by a pressure (or chemical potential) difference between the vapor and liquid phases. This model assumes that molecular kinetic theory applies, no bulk flow across the interface (quiescent conditions), local thermodynamic equilibrium on each side of the interface, and a flat interface with no curvature effects (surface tension neglected).

The Hertz–Knudsen–Langmuir (HKL) model is a simplified form of the Hertz–Knudsen equation, applicable primarily to pure evaporation or high-vacuum conditions, where back condensation is negligible. Following Langmuir [53], the mass flux reduces to a one-sided evaporation rate,

(9) $${\overset{˙}{m}}_{\mathrm{HKL}}=\alpha \sqrt{\frac{M}{2\pi RT}}\;p_{\mathrm{sat}}\left(T\right),$$

which assumes that all molecules leaving the interface escape into the vapor phase without recondensing. The HKL model further assumes a steady interface temperature and local thermodynamic equilibrium between the interface and the vapor phase, making it unsuitable for situations where evaporation and condensation occur simultaneously. To overcome these limitations, Schrage [54] proposed an extended formulation, commonly referred to as the Hertz–Knudsen–Schrage (HKS) model. This model accounts for nonequilibrium conditions across the liquid–vapor interface by allowing for differences in temperature and pressure between the phases and by considering bidirectional mass transfer. The net interfacial mass flux is commonly written as

(10) $${\overset{˙}{m}}_{\mathrm{HKS}}=\frac{2\alpha }{2-\alpha }\sqrt{\frac{M}{2\pi R}}\left(\frac{p_{\mathrm{sat}}\left(T_{l}\right)}{\sqrt{T_{l}}}-\frac{p_{v}}{\sqrt{T_{v}}}\right),$$

where $T_{l}$ and $T_{v}$ denote the liquid and vapor temperatures at the interface, respectively. Compared to the HKL formulation, the HKS model enables the simultaneous modeling of evaporation and condensation and captures nonequilibrium thermal effects, which are particularly important in rapidly evolving flows such as boiling and cavitation.

In cavitating flows, particularly those involving compressibility, the pressure and temperature near the bubble interface can deviate significantly from thermodynamic equilibrium. Under these nonequilibrium conditions, the HKS model provides a more accurate representation of the phase transition process than simpler models like HKL. It captures the dynamic interplay between evaporation and condensation, especially during rapid bubble expansion and violent collapse. The model is supposed to be suited for scenarios involving rapid pressure fluctuations, where the direction and magnitude of mass transfer across the interface change continuously, [54]. The HKS model is built upon several important assumptions. First, it assumes that local thermodynamic equilibrium exists within each phase, meaning that both the liquid and vapor phases are individually in equilibrium, but not necessarily across the interface. Second, the model relies on the Maxwellian velocity distribution of molecules near the interface, consistent with kinetic theory. Finally, it incorporates a finite accommodation coefficient, which accounts for the probability that a molecule striking the interface will successfully undergo phase transition (either condense or evaporate), and needs to be estimated.

Another group of phase transition models consists of empirical-semi-theoretical models used to simulate macroscopic cavitation in liquid flows. These models treat cavitation as a mass transfer process between liquid and vapor phases, driven by local pressure conditions and bubble dynamics. A widely used representative of this group is the Schnerr–Sauer model, [55]. A detailed description of this approach is given in the following paragraph.

A further class of phase transition models consists of barotropic formulations, in which phase change occurs instantaneously under thermodynamic equilibrium conditions. In these models, the pressure is prescribed as a function of density through a barotropic equation of state, and effects related to nuclei size, number, or distribution are neglected [56], [57].

All models described above rely on simplifying assumptions and, in many cases, empirical parameters. For example, Hertz–Knudsen-based models require the specification of an accommodation coefficient, while empirical-semi-theoretical models such as Schnerr–Sauer depend on parameters related to bubble population characteristics. In the present investigation, we employ both a Hertz–Knudsen-based model, namely the formulation of Phan et al. [58], [27] extended by Peters and el Moctar [59], and the empirical-semi-theoretical Schnerr–Sauer model. Although the Schnerr–Sauer model is not theoretically valid for single-bubble dynamics, its widespread use motivates an assessment of its applicability under such conditions. The extended Phan et al. model is based on the theory of critical phenomenon dynamics, as explained by Hohenberg and Halperin [60], Lee [61]. The theory assumes that the mass transfer rates depend on the difference between value of a flow quantity and its critical value. While Lee [61] used the difference between the actual and critical temperatures at which the phase transition begins to calculate non equilibrium phase transition processes in a two-phase mixture, Phan et al. used the difference between the absolute and vapor pressures. This approach is analogous to the Hertz–Knudsen group of models.

In the current study phase transition between vapor and liquid is modeled using a pressure-based non-equilibrium formulation of Phan et al. [58], [27] and extended by Peters and el Moctar [59]. The mass transfer rates for condensation and vaporization per unit volume are defined as follows:

(11) $${\overset{˙}{m}}_{c}=c_{cp}\alpha_{l}\left(1-\alpha_{l}\right)\rho_{l}\frac{\max\left(p-p_{\mathrm{sat}},0\right)}{p_{\mathrm{sat}}},$$

(12) $${\overset{˙}{m}}_{v}=c_{vp}\alpha_{l}\left(1+\alpha_{\mathrm{nuc}}-\alpha_{l}\right)\rho_{l}\frac{\min\left(p-p_{\mathrm{sat}},0\right)}{p_{\mathrm{sat}}},$$

where $c_{cp}$ and $c_{vp}$ are the coefficients of condensation and vaporization, respectively, $\alpha_{nuc}$ is the volume fraction of vapor nuclei, assumed as 1e-6, and $p_{\mathrm{sat}}$ is the saturation pressure. The Schnerr–Sauer cavitation model [55] is also employed to describe the phase transition. It accounts for non-equilibrium phase change by defining vaporization and condensation based on bubble dynamics and the distribution of nuclei, assuming that the vapor phase is represented by the cumulative volume of single bubbles per unit liquid volume. The mass transfer rates for condensation and vaporization per unit volume is defined as follows:

(13) $${\overset{˙}{m}}_{c}=c_{cs}\;\frac{3\;\alpha_{l}\;\rho_{l}\rho_{v}}{\rho \;R_{b}}\;\max\left(p-p_{\mathrm{sat}},0\right)\;\sqrt{\frac{2}{3\;\rho_{l}\;\left|p-p_{\mathrm{sat}}\right|}}$$

(14) $${\overset{˙}{m}}_{v}=c_{vs}\;\left(1+\alpha_{\mathrm{nuc}}-\alpha_{l}\right)\;\frac{3\;\alpha_{l}\;\rho_{l}\rho_{v}}{\rho \;R_{b}}\min\left(p-p_{\mathrm{sat}},0\right)\;\sqrt{\frac{2}{3\;\rho_{l}\;\left|p-p_{\mathrm{sat}}\right|}}$$

where $c_{cs}$ and $c_{vs}$ are the coefficients of condensation and vaporization, respectively. The coefficients $c_{cs}$, $c_{vs}$, $c_{cp}$, and $c_{vp}$ were calibrated against experimental data for γ=2.0, using the maximum equivalent bubble radius after the first collapse (rebound radius) as the reference metric, and then applied consistently to all simulations. To assess robustness, a sensitivity analysis was performed by varying the condensation and vaporization rate coefficients separately: first, the condensation rate coefficient was varied while keeping the vaporization coefficient constant, and second, the vaporization rate coefficient was varied while keeping the condensation coefficient constant.

$R_{b}$ is the radius of the cavitation nuclei, expressed as follows

(15) $$R_{b}={\left(\frac{1-\alpha_{l}-\alpha_{nuc}}{\alpha_{l}}\;\frac{3}{4\pi n}\right)}^{\frac{1}{3}}$$

Here $\alpha_{\mathrm{nuc}}=\frac{n\pi 4/3R_{0}^{3}}{1+n\pi 4/3R_{0}^{3}}$, is the volume fraction of vapor nuclei, where $R_{0}$ is the initial nucleus radius ($0.5\times 10^{-15}\;\text{m}$) and n is the nuclei number density per cubic meter ($1.0\times 10^{20}\;{\text{m}}^{-3}$ in our simulation). The saturation pressure is defined as a function of temperature using the August–Roche–Magnus relation [62], which reads as follows:

(16) $$p_{\mathrm{sat}}\left(T\right)=C_{1}\exp\;\left(\frac{C_{2}T}{C_{3}+T}\right),$$

where T is the temperature in K. $C_{1}$, $C_{2}$, and $C_{3}$ are empirical constants for liquid water.

The temperature field is computed from the compressible energy equation, which includes pressure work and thermal conduction. The governing energy equation reads as follows:

(17) $$\frac{\partial \rho T}{\partial t}+\nabla \cdot \left(\rho uT\right)-\nabla \cdot \left(\beta \nabla T\right)=$$

$$\left(\frac{\alpha_{l}}{C_{v,l}}+\frac{1-\alpha_{l}}{C_{v,v}}\right)\left[\left({\overset{˙}{m}}_{c}+{\overset{˙}{m}}_{v}\right)H_{v}-\frac{\partial \rho k}{\partial t}-\nabla \cdot \left(\rho uk\right)-\nabla \cdot \left(up\right)\right]$$

where $k=0.5{\left|u\right|}^{2}$ is the kinetic energy per unit mass. Here β is the thermal diffusivity of the mixture and is given by $\alpha_{v}\beta_{v}+\alpha_{l}\beta_{l}$. $C_{v,v}$ and $C_{v,l}$ are the specific isochoric heat capacities of the vapor and liquid phases, respectively, and $H_{v}$ is the latent heat of vaporization that accounts for temperature variations due to phase transition [59]. Recall that condensation leads to an increase in temperature, whereas vaporization has the opposite effect.

The compressibility of each phase is modeled via appropriate equations of state. The liquid phase follows the Tait equation:

(18) $$\rho_{l}=\rho_{\mathrm{ref}}{\left(\frac{B+p}{B+p_{0}}\right)}^{1/n},$$

where $\rho_{\mathrm{ref}}=1000\;{\mathrm{kg/m}}^{3}$, $B=3.046\times 10^{7}\;\mathrm{Pa}$, $p_{0}=10^{5}\;\mathrm{Pa}$, and n=7.1. The vapor density is calculated using the ideal gas equation:

(19) $$\rho_{v}=\frac{p}{R_{v}T},$$

with $R_{v}$ is the specific gas constant for the vapor. While commonly used in cavitation simulations, the ideal gas assumption may become inaccurate at the very high pressures reached during collapse (several thousand bar), where real-gas effects can influence the thermodynamic response. This limitation will be addressed in future work using real-gas equations of state and inclusion of non-condensable gas.

**Fig. 1:**
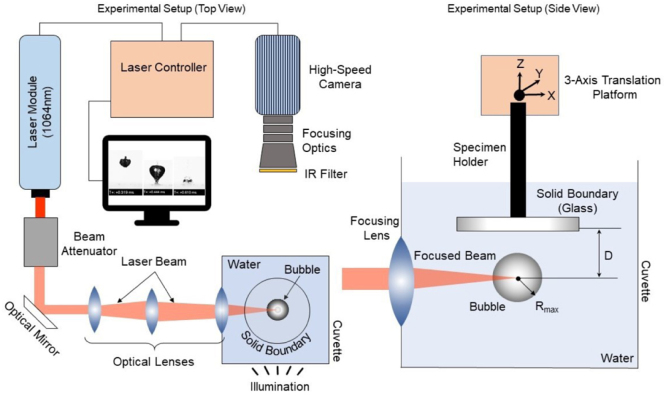
Experimental setup employed to generate a laser-induced single cavitation bubble near a solid boundary.

## 3 Experimental method

The laser-induced bubble collapse experiments were based on our previously developed setup of Sagar and el Moctar [63]. The setup consisted of a Q-switched Nd:YAG laser, optical components for beam focusing and attenuation, a high-speed imaging system, and a transparent cuvette filled with tap water. All components were mounted on a vibration-isolated optical table.

The cuvette, made of transparent acrylic (50 mm × 50 mm ×50 mm of 10 mm wall thickness), was filled with water. A one-inch focusing lens embedded in one wall focused the laser beam on an oppositely placed optical window to avoid wall damage. A single distinct cavitation bubble was generated by focusing a laser pulse (wavelength 1064 nm, 6 ns duration, max 450 mJ energy) into the liquid, where it created a plasma and a vapor bubble. A beam attenuator and wedge prism reduced the beam energy to between 18 and 22 mJ to control the bubble’s size, which typically had a radius of around 3.0 ± 0.1 mm.

Bubble dynamics were captured using a Phantom v2012 high-speed camera equipped with a CMOS sensor with a pixel length of 28  μ m and a 200 mm Micro-Nikkor lens. The imaging system used a 60 W diffused LED light source in a back-illumination technique, and an infrared filter protected the sensor from laser scatter. Calibration was performed using a millimeter grid to convert pixels to physical units. The camera was focused on the bright region at the center of the bubble shadow, which served as the bubble center. The experiments were conducted using tap water, and the dissolved gas content was not explicitly controlled. This may influence cavitation dynamics and is acknowledged as a limitation of the present study.

A 15 mm diameter, 3 mm thick glass plate, used as a solid boundary, was mounted on a three-axis translation stage. By adjusting its vertical position, the target stand-off distance was obtained. The glass was aligned with the plasma center before each measurement. Fig. 1 shows the experimental setup. Experimental uncertainties, including fluctuations in laser energy, local variations in water properties, and impurities, introduce a variability of approximately 3% in bubble size. For γ=1.0 and γ=2.0, the deviations are about 8.8% for the first collapse time, 3.7% for the minimum radius, and 5.7% for the rebound amplitude.

## 4 Numerical setup

The simulations were conducted using an open-source finite volume-based solver for compressible two-phase flows, which we extended to account for mass transfer and latent heat.

**Fig. 2:**
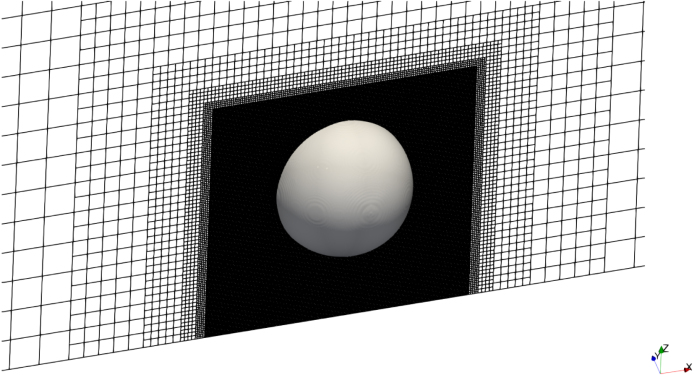
Numerical grid with refined region surrounding the bubble.

**Fig. 3:**
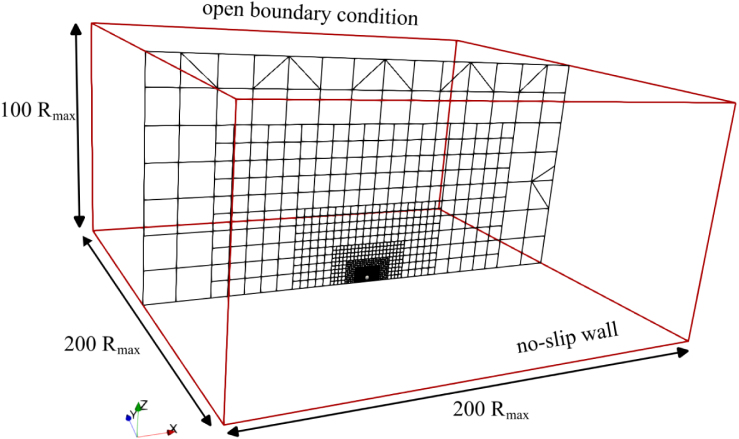
Computational domain and boundary conditions.

The computational domain comprised a three-dimensional box of dimensions $200\;\times 200\;\times 100\;R_{\max}$, where $R_{\max}$ is the maximum bubble radius. The domain contained about 20.7 million control volumes (CVs). A locally refined region of $4\times 4\times 2\;R_{\max}$ surrounded the bubble to effectively resolve interface dynamics and gradients. The minimum CV size in this region was denoted as $\Delta x_{\min}$. Fig. 2 shows a close-up view of this locally refined region and Fig. 3 the entire computational domain with the applied boundary conditions.

The simulation was initialized with a fully grown vapor bubble of 1.5 mm radius. The surrounding liquid was at ambient pressure $p_{\infty }=0.101\;\text{MPa}$ and temperature T=293.15K, and it was not initially flowing. The vapor volume fraction was set to $\alpha_{\text{vapor}}=1.0$ inside the bubble and to zero outside the bubble. At the solid boundary, the no-slip conditions were applied. Zero-gradient conditions were applied to both pressure and temperature. At the outlet boundary an open boundary condition was applied for velocity, zero-gradient temperature, and volume fraction. Time integration was performed using the first-order implicit Euler scheme. To ensure numerical stability and resolution of fast transients, the time step was adaptively controlled by the acoustic Courant number, here constrained to $CFL_{\text{acoustic}}\leq 0.1$, as it was related to the speed of sound rather than the flow velocity. The smallest time step reached during simulations was $\Delta t=4.5\times 10^{-10}\;\text{s}$. Pressure-velocity coupling was accounted for using the PIMPLE algorithm. Stopping the outer iterations was based on a prescribed convergence criterion. All the spatial discretization schemes employed were of second-order accuracy.

## 5 Verification and validation

### 5.1 Discretization and grid convergence study

To quantify discretization uncertainty and evaluate the influence of mesh resolution on the simulation of a cavitation bubble collapsing near a rigid wall, we carried out a discretization study. All simulations were conducted at a stand-off distance of γ=2.0, a configuration that induced noticeable bubble deformation and a non-spherical shape at the end of the first collapse, followed by an evident bubble rebound.

The evaluated bubble dynamics were obtained on three systematically refined meshes. In all meshes, the physical model and the numerical schemes remained identical. Only the grid resolution and the time step were varied. The coarsest mesh comprised 12.5 million control volumes; the medium mesh, 20.7 million control volumes; the finest mesh, 35.2 million control volumes. The refinement factor between successive meshes was $\sqrt{2}$. Table 1 summarizes the results obtained on the three grids as well as the extrapolated results.

The method proposed by Oberhagemann [64], el Moctar et al. [65] was employed to combine spatial and temporal refinements into a single nondimensional parameter, known as the refinement ratio $\Upsilon_{i}$ with index i=1,2, and 3 corresponding to the coarse, the medium, and the fine meshes, respectively:

(20) $$\Upsilon_{i}=\sqrt{\frac{1}{4}\left[{\left(\frac{1}{r_{x}}\right)}^{2\left(i-1\right)}+{\left(\frac{1}{r_{y}}\right)}^{2\left(i-1\right)}+{\left(\frac{1}{r_{z}}\right)}^{2\left(i-1\right)}+{\left(\frac{1}{r_{t}}\right)}^{2\left(i-1\right)}\right]}$$

In this study, the refinement factors in the x, y, z directions and at time t were constant: $r_{x}=r_{y}=r_{z}=r_{t}=\sqrt{2}$.

**Table 1:** Results of the discretization study.

Grid	Number of control volumes	Acoustic CFL	$\Upsilon_{i}$	$R_{\text{max,1}}$	$p_{\text{max}}$	$T_{\text{max}}$
	[million]	[–]	[–]	[–]	[bar]	[K]
Coarse	12.5	0.1	1.00	0.619	219.5	451.4
Medium	20.7	0.1	0.71	0.626	248.5	455.4
Fine	35.2	0.1	0.50	0.628	280.4	472.2

Extrapolated	–	–	–	0.631	294.9	474.2

Evaluated quantities comprised the maximum normalized equivalent bubble radius $R_{\text{max},1}$ after the first collapse, the maximum pressure $P_{\text{max}}$ on the wall, and the maximum temperature $T_{\text{max}}$ inside the bubble. The equivalent bubble radius was normalized against the maximum bubble radius $R_{\text{max}}$ at the time instance of initialization. The computed values $\phi_{i}$ were fitted to a second-order polynomial:

(21) $$\phi_{i}=\phi_{\infty }+a_{1}\cdot \Upsilon_{i}+a_{2}\cdot \Upsilon_{i}^{2},\;i=1,2,3$$

where $\phi_{\infty }$ denotes the extrapolated grid-independent solution, obtained via least-squares minimization.

**Fig. 4:**
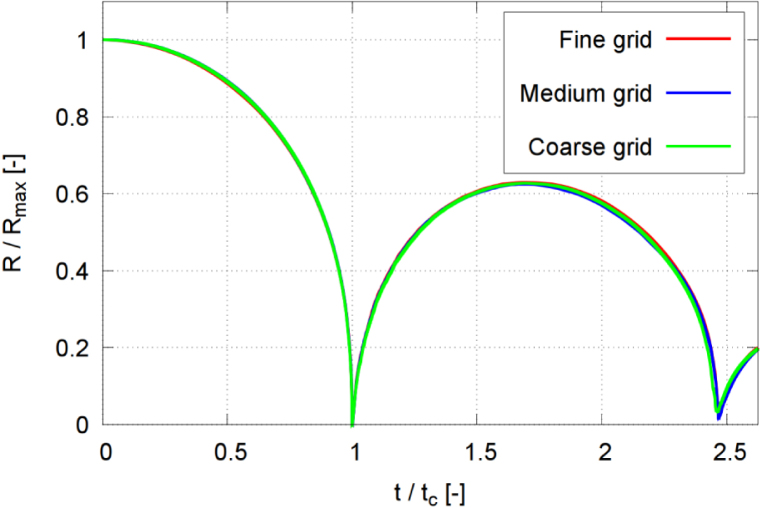
Normalized equivalent bubble radius $R/R_{\text{max}}$ in each time step, obtained on the three successively refined grids for γ=2.0, and with time t normalized against collapse time $t_{c}$.

**Fig. 5:**
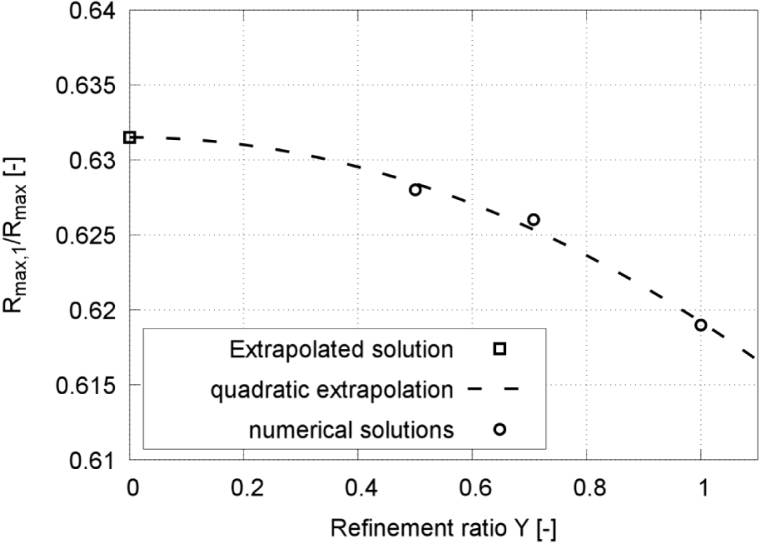
Extrapolated discretization-independent maximum bubble radius $R_{\text{max},1}$ vs. refinement ratio Υ, obtained from the extrapolated solution, the quadratic extrapolation, and the numerical simulation, for γ=2.0.

**Fig. 6:**
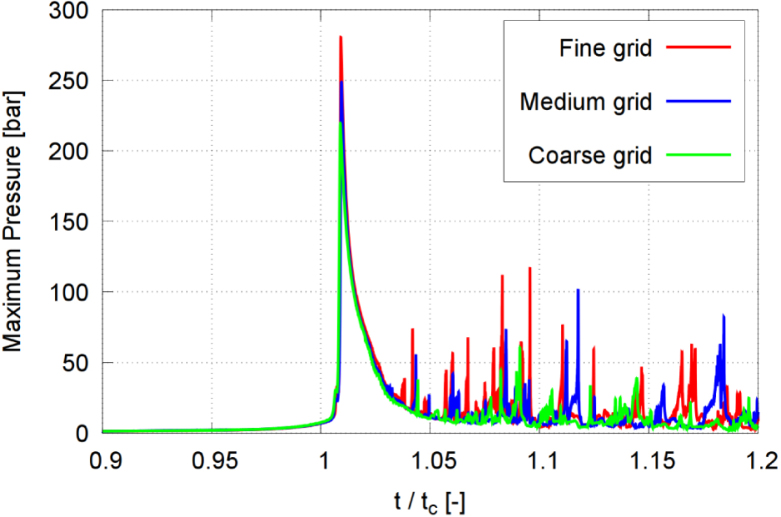
Maximum pressure on the wall in each time step, obtained on the three successively refined grids for γ=2.0, and with time t normalized against collapse time $t_{c}$.

**Fig. 7:**
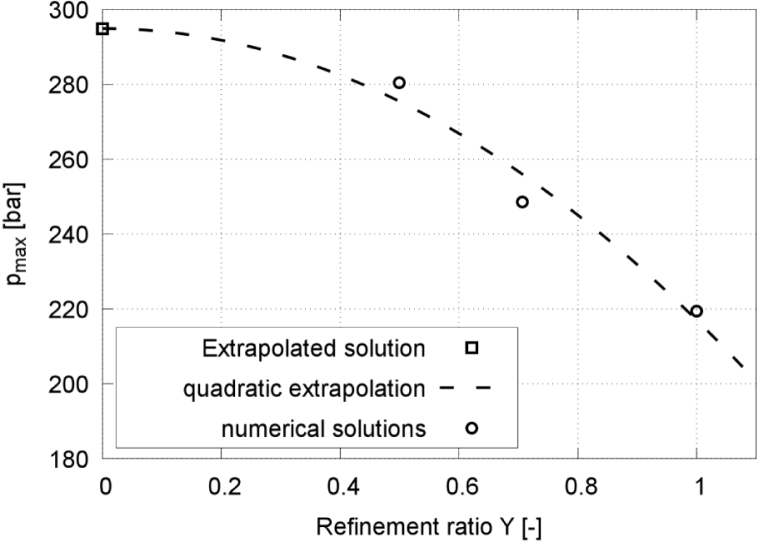
Extrapolated discretization-independent maximum pressure $P_{\text{max}}$ on the wall vs. refinement ratio Υ, obtained from the extrapolated solution, the quadratic extrapolation, and the numerical simulation for γ=2.0.

**Fig. 8:**
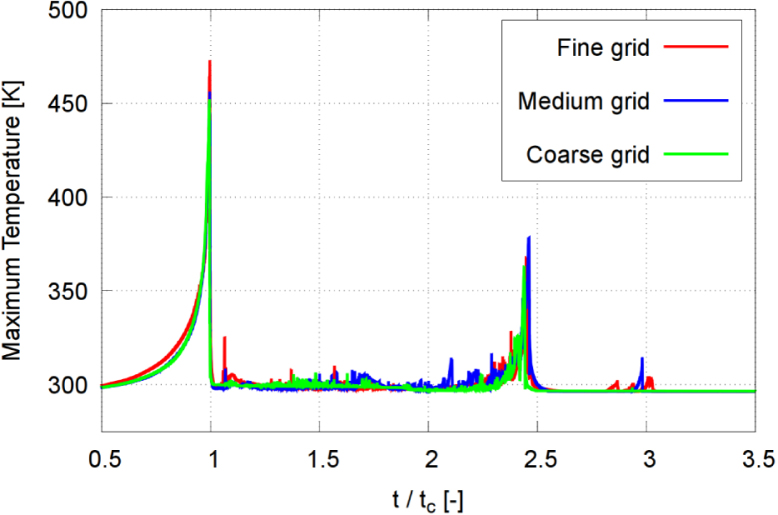
Maximum temperature inside the bubble in each time step, obtained on the three successively refined grids for γ=2.0, and with time t normalized against collapse time $t_{c}$.

**Fig. 9:**
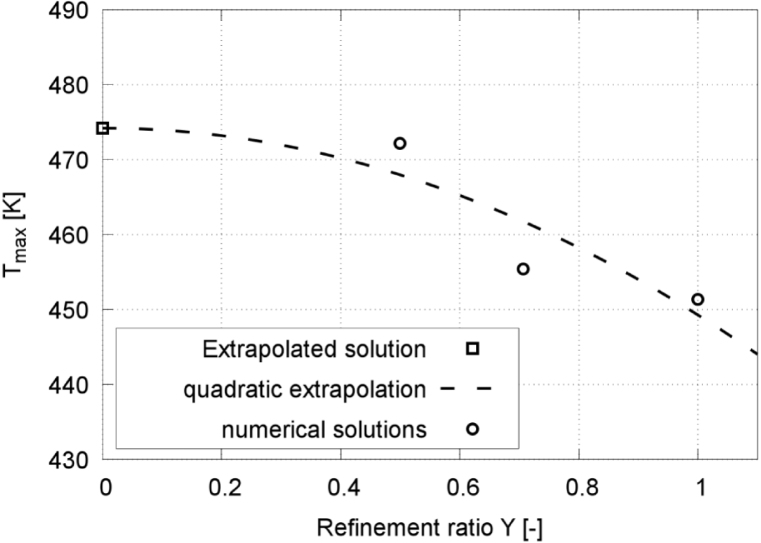
Extrapolated discretization-independent maximum temperature $T_{\text{max}}$ vs. dimensionless refinement ratio Υ, obtained from the extrapolated solution, the quadratic extrapolation, and the numerical simulation for γ=2.0.

Fig. 4, Fig. 6 present time histories of the normalized equivalent bubble radius $R/R_{\text{max}}$ and the maximum pressure on the wall, respectively, obtained on the fine (red line), medium (blue line), and coarse (green line) meshes. Fig. 5 plots the converging extrapolated discretization-independent maximum bubble radius $R_{\text{max},1}$ at rebound, obtained from the extrapolated solution (squares), the quadratic extrapolation (dashed line), and the numerical simulation (circles), versus refinement ratio Υ. Fig. 7 plots the extrapolated discretization-independent maximum pressure $P_{\text{max}}$ on the wall, obtained from the extrapolated solution (blue squares), the quadratic extrapolation (dashed line), and the numerical simulation (black dots), versus refinement ratio Υ. Fig. 8 presents time histories of the maximum temperature inside the bubble, and Fig. 9 plots the converging behavior of the temperature peak $T_{\text{max}}$, both obtained from the extrapolated solution (squares), the quadratic extrapolation (dashed line), and the numerical simulation (circles).

The normalized maximum bubble radius ratio after the first collapse converged monotonically, increasing from 0.619 to 0.628, obtained on the coarse and the fine mesh, respectively, whereby the extrapolated value was 0.629. The values obtained on the coarse and medium grids deviated by 1.4% and 0.3%, respectively, from those obtained on the fine grid.

The maximum pressure obtained on the fine grid reached 280.4 bar, which was 11.4% and 21.7% higher than the maximum pressure obtained on the medium grid and the coarse grid, respectively. The maximum temperature obtained on the fine grid reached 472 K, which was 3.6% and 4.4% higher than the maximum temperature obtained on the medium grid and the coarse grid, respectively. Pressure and temperature, however, exhibited a higher sensitivity to mesh resolution, reflecting the compressible and transient nature of the collapse event. Based on accuracy and efficiency, we decided to perform all subsequent simulations on the medium grid. The physical shock thickness in water is on the order of O(1μm) [66], which is significantly smaller than the present grid spacing (Δx=21μm). Therefore, while the simulations capture shock propagation, the internal shock structure is not fully resolved and peak pressures should be interpreted as resolution-dependent upper bounds.

To further assess grid convergence, the convergence ratio was evaluated as

(22) $$r_{conv}=\frac{\phi_{2}-\phi_{1}}{\phi_{3}-\phi_{2}},$$

where $\phi_{1}$, $\phi_{2}$, and $\phi_{3}$ denote the solutions obtained on the fine, medium, and coarse grids, respectively. Based on Table 1, the convergence ratio for the maximum rebound radius was found to be $r_{conv}=0.29$, indicating monotonic convergence. Furthermore, key global quantities such as collapse timing and rebound dynamics show consistent behavior across grid levels, supporting the use of the medium grid as a reasonable compromise between accuracy and computational cost.

### 5.2 Validation

To validate the numerical model, we compared our numerical results with our experimental measurements, where the bubble’s dynamic behavior was monitored at a dimensionless stand-off distance of γ=2.0, a distance that at which different scenarios like jet formation, rebound, and a second collapse were expected to occur.

**Fig. 10:**
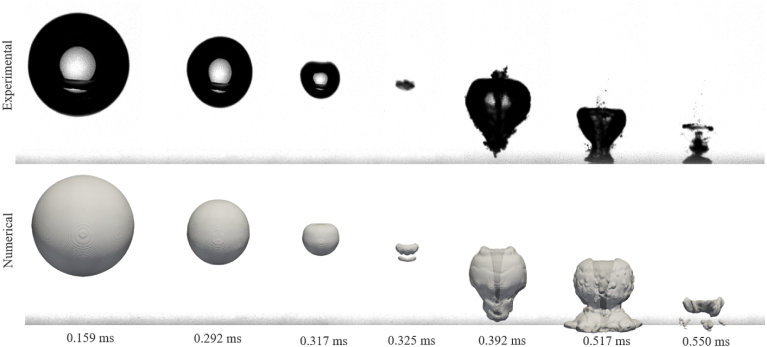
Sequential bubble dynamics, obtained from experiments and numerical simulation, for γ=2.0 at times t=0.159, 0.292, 0.317, 0.325, 0.392, 0.517, and 0.550ms.

Fig. 10 shows the temporal evolution of bubble shapes obtained from the experiments and the numerical simulation, beginning with the bubble’s fully grown size to the end of its second collapse. The upper and lower sequences display images obtained from the physical experiments and the numerical simulation, respectively, at matching time steps. The maximum bubble radius reached in both sequences was about 1.5 mm. As seen, the bubble’s dynamics, including its collapse, and rebound, generally compared favorably.

Jet formation became visible at about t=0.317ms, and the bubble rebound began shortly after at t=0.325ms. The experimentally determined direction and structure of the jet also compared favorably to those from the numerical simulation. In both cases, the lower interface narrowed during collapse, and the jet penetrated towards the wall. The bubble shapes clearly show a focusing region along the jet axis, consistent with the experimental recordings.

**Fig. 11:**
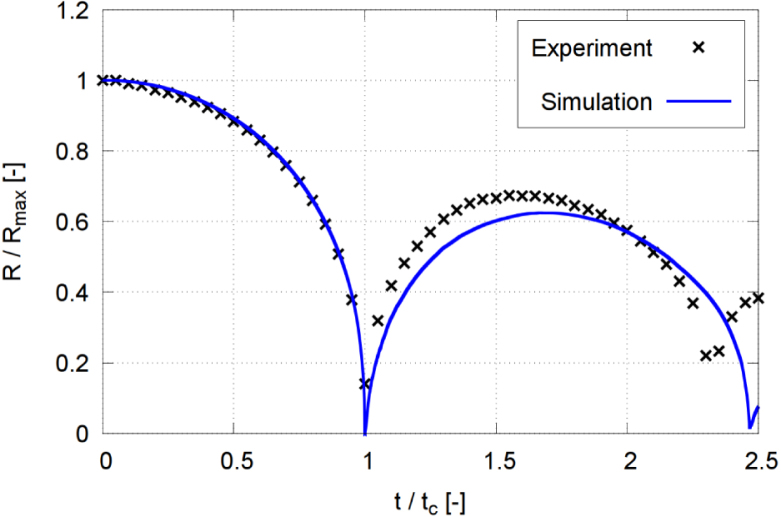
Normalized equivalent bubble radius $R/R_{\text{max}}$ in each time step, obtained from experiments and numerical simulation for γ=2.0, and with time t normalized against collapse time $t_{c}$.

Fig. 11 presents comparative time histories of the normalized equivalent bubble radius $R/R_{\text{max}}$, starting from the fully grown bubble, obtained from experiments (black crosses) and simulation (blue line). As seen, during the bubble’s first oscillation, the simulated and the experimental radius matched accurately. After rebound, the bubble’s radius deviated by about 14%, with its equilibrium radius in the simulation being slightly smaller. This difference may have been due to the two-dimensional nature of the photographs, considering that the bubble shape captured in the experiment and the bubble area could only be estimated. Consequently, the bubble volume tended to be overestimated, especially when the jet hit the bubble and generated its torus-like shape. In contrast, the bubble’s numerically predicted equilibrium radius was based on the fully three-dimensional vapor volume. Overall, the comparison demonstrated that the implemented model, which included phase transition, and compressibility in a full three dimensional flow resolution, was capable to reproduce the main physical mechanisms governing bubble collapse near a solid wall. Furthermore, the numerical framework employed in the present study has previously been applied and validated for shock-dominated compressible two-phase flows and sloshing-induced impact pressures using similar spatial resolutions [59].

### 5.3 Comparison of phase transition models

We compared the equivalent bubble radius obtained by two different phase transition models against the experimental results. The model of Phan et al. [27] extended by Peters and el Moctar [59] (Eqs. (11), (12)) is a pressure-based model that specifically describes single-bubble cavitation rather than cavitating flow. On the other hand, the Schnerr–Sauer model of Sauer and Schnerr [55] is a traditional approach for handling macroscopic cavitating flows. Several authors used the Schnerr–Sauer model as a phase transitional method to study single-bubble cavitation [63], [67].

**Fig. 12:**
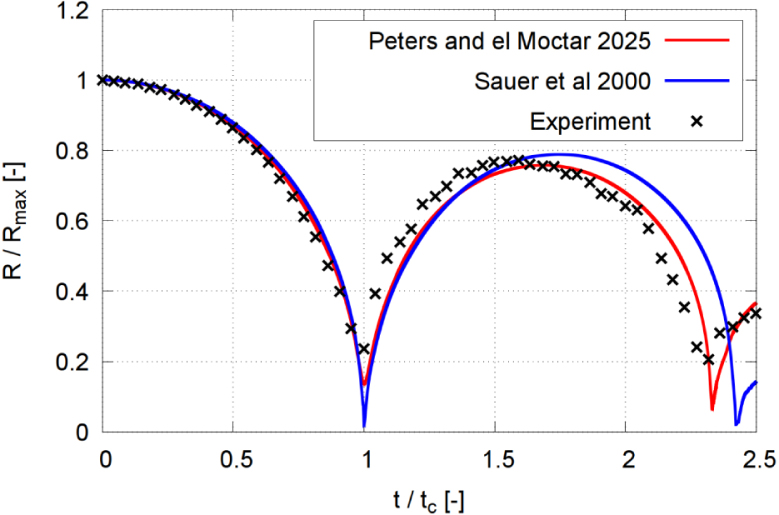
Time histories of the normalized equivalent bubble radius $R/R_{\text{max}}$, obtained from experiments and simulations using the models of Phan et al. [27] extended by Peters and el Moctar [59], Sauer and Schnerr [55], for γ=1.0, $c_{cp}=2\;{\text{s}}^{-1}$ and $c_{vp}=5\;{\text{s}}^{-1}$ with the model of Peters and el Moctar [59], $c_{cs}=10$ and $c_{vs}=10$ with the model of Sauer and Schnerr [55], and with time t normalized against collapse time $t_{c}$.

Fig. 12 plots comparative time histories of the normalized equivalent bubble radius $R/R_{\text{max}}$ obtained from experiments (black crosses) and simulations using the extended Phan et al. model (red line) and the Schnerr–Sauer model (blue line). The simulations started from the fully grown bubble. As seen, both models predicted acceptable agreement with the experimental results, especially during the bubble’s first collapse. During the first oscillation cycle, the measured and computed time histories of the equivalent bubble radius compared favorably. However, during the second oscillation cycle, the differences became more pronounced. Here, the extended Phan model predicts the timing of collapse and rebound events with greater accuracy. Generally, predictions with both models were similar and compared favorably to the experiments. This showed that, although the Schnerr–Sauer model was originally formulated only for macroscopic cavitation and cavitation flow, it also reproduced the dynamics of single bubble cavitation.

## 6 Results

We started our numerical simulations for a single cavitation bubble collapsing near a solid wall with a fully grown bubble beginning its first collapse phase. The influence of the wall was defined by the stand-off distance $\gamma =D/R_{\text{max}}$, where D is the distance between the bubble center in its initial condition to the wall. We considered the two stand-off distances γ=1.0 and 2.0 to represent strong to weak wall effects.

### 6.1 3D flow surrounding the collapsing bubble

#### 6.1.1 Velocity field

When the bubble attained its maximum size, the interface velocity was almost zero, and the pressure inside the bubble was close to vapor pressure. The external pressure pushed the flow inward towards the bubble center. The bubble’s first oscillation cycle (first collapse phase) was primarily inertia driven. Although phase transition did not play a crucial role during this cycle, condensation might still have led to mass loss. After the first collapse, the effect of phase transition became crucial and started to dominate bubble dynamics.

**Fig. 13:**
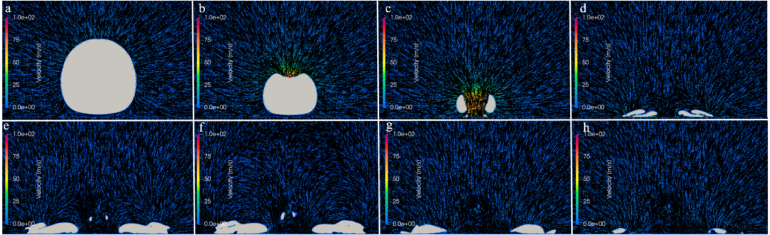
For γ=1.0, successive time frames of velocity fields surrounding the bubble during its first collapse until the end of its second collapse. Frame a shows the bubble shortly after its maximum size ($T_{\text{ini}}+0.04\;\mathrm{ms}$); frame b, during its first collapse and jet penetration ($T_{\text{ini}}+0.13\;\mathrm{ms}$); frames c and d, at the last moment of the first collapse ($T_{\text{ini}}+0.15\;\mathrm{ms}$) and the beginning of its rebound ($T_{\text{ini}}+0.18\;\mathrm{ms}$); frames e and f, during its rebound ($T_{\text{ini}}+0.24$ and 0.28ms); and frames g and h, during its second collapse ($T_{\text{ini}}+0.37$ and 0.40ms).

**Fig. 14:**
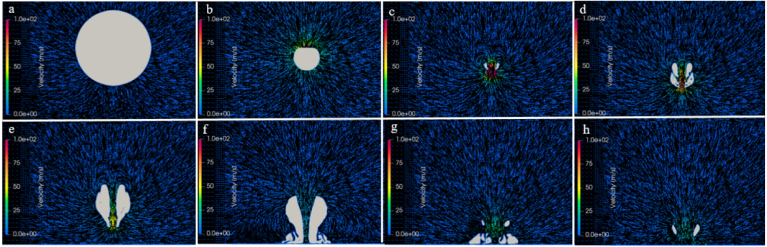
For γ=2.0, successive time frames of velocity fields surrounding the bubble during its first collapse until the end of its second collapse. Frame a shows the bubble fully grown ($T_{\text{ini}}=0\;\mathrm{ms}$); frames b, during its first collapse and jet penetration ($T_{\text{ini}}+0.14\;\mathrm{ms}$); frame c, at the last moment of the first collapse and the beginning of its rebound ($T_{\text{ini}}+0.15\;\mathrm{ms}$); frames d, e, and f, during its rebound ($T_{\text{ini}}+0.16$, 0.18, and 0.28ms); and frames g and h, at its second collapse ($T_{\text{ini}}+0.35$ and 0.37ms).

For γ=1.0 and γ=2.0, Fig. 13, Fig. 14 each present eight successive frames of so-called velocity fields that identify the flow surrounding the bubble during its first collapse until the end of its second collapse. The small-time intervals between these frames were measured from the initial time $T_{ini}=0$ s of the bubble’s generation. The velocity fields show distributed, colored velocity vectors projected on a vertical plane cutting through the bubble in the x-direction. The vectors’ orientation specify flow directions; the vectors’ color, flow magnitudes, identified by attached color bars ranging from 0 to 100 m/s.

Fig. 13 shows the velocity fields for the bubble positioned in close proximity to a solid boundary, i. e., for γ=1.0. The collapse time determined by the simulation was $t_{c}=0.178\;\mathrm{ms}$. It is well known that, when the stand-off distance is less than unity, the bubble grows non-spherically. To take this into account, the growth phase was simulated based on the method described in Section 6.2.1. Frame a shows the non-spherical bubble shortly after it was fully grown at its maximum size. Its lower side, adjacent to the wall, flattened and took on the wall shape. Frame b, 0.13 ms later, shows the jet starting to penetrate the bubble during its first collapse. Field c, 0.15 ms, shows the bubble at the end of its first collapse, where the jet fully penetrated the bubble and hit the wall directly. At this point, the maximum velocity of the water jet was about 207 m/s. Although not directly obtainable from the contour of distributed velocities, this velocity was extracted from the distributed velocity vectors. From now on, a toroidal vapor structure developed, a characteristic feature under conditions of strong phase transition and flow asymmetry. The velocity vectors near the boundary were redirected outward. At the same time, the surrounding liquid accelerated inward towards the bubble center. The bubble began to rebound, adopting an annular shape due to its asymmetric collapse, as shown in frames d and e. Frame f shows the beginning of the bubble’s second collapse. Here, the maximum bubble radius was noticeably (21%) smaller than during the previous (first) collapse, which was clearly indicated the reduced amount of vapor. In frames g and h, the velocities flowed outward, caused by the reflecting flow reversal.

Fig. 14 shows the velocity fields for the bubble positioned an increased distance from the wall, i. e., for γ=2.0. By increasing this stand-off distance, the effect of the wall on the bubble’s collapse decreased. This also led to a reduced collapse time of $t_{c}=0.157\;\mathrm{ms}$. Frames b and c show that, at the end of the first collapse, a jet started to form and deformed the bubble. The collapse was faster compared to the previous case (γ=1.0). Now, the jet entirely penetrated the bubble as it reached its minimum size during the first collapse. The jet caused the bubble to stretch further in the vertical z-direction. The jet velocity reached around 237 m/s. This strong downward motion expanded the lower part of the bubble, causing it to form a narrow, elongated shape. It continued as the bubble rebounded until hitting the solid boundary. Due to the velocity gradient between the fast-moving jet and the slower upper part, the bubble’s bottom part became detached from its upper part. As this detached bottom part separated, the upper part continued to shrink, as seen in frame g. This shows that, even for this somewhat larger stand-off distance, the local flow was affected as seen by the jet being stretched. The increase in jet velocity with stand-off distance is attributed to reduced wall-induced confinement: for small γ, geometric restriction limit jet development, whereas for larger γ, a more symmetric collapse allows sustained axial acceleration and higher jet velocities, consistent with the trends reported by Koch et al. [68] and Bußmann et al. [69].

#### 6.1.2 Pressure wave

We numerically simulated the pressure field during the first and second oscillation cycles as well as the resulting pressure acting on the wall. At the last stage of the first collapse, when reaching the minimum equivalent radius $R_{\text{min}}$, the condensation led to a violent flow, characterized by high pressure, velocity, and temperature. From the energy released at this time, a shock wave was generated. The emitted shock wave was centralized at the bubble and propagated into the surrounding liquid. In a free velocity field, it traveled spherically; however, placing a solid boundary in the vicinity of the bubble interfered with its dynamics. Part of the energy not transferred to the shock wave contributed to the formation of a jet. When placed further from the wall, the bubble’s collapse became more spherical, and the associated shock wave was stronger [70]. However, the pressure wave arriving at the wall was weakened.

**Fig. 15:**
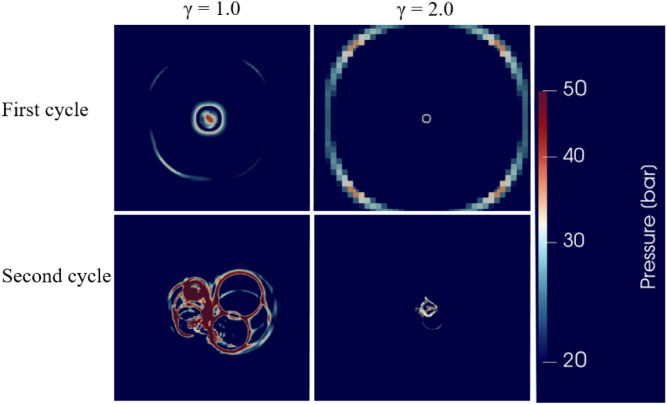
In a region representing an area of $7R_{\text{max}}\times 7R_{\text{max}}$ surrounding the collapsing bubble, distributed pressures, determined for the maximum pressure wave heading towards the solid boundary, caused by an oscillating bubble for γ=1.0 and γ=2.0 during its first and second collapses. At 1.61μs after the first collapse and 1.0μs after the second collapse, the maximum pressures for γ=1.0 are 41.83 bar and 888.24 bar, respectively. For γ=2.0, at 4.52μs and 3.58μs after the first and second collapses, the corresponding pressures are 35.77 bar and 101.71 bar.

For γ=1.0, the bubble collapse was more violent compared to a greater γ. In a region representing an area of $7R_{\text{max}}\times 7R_{\text{max}}$ (top view) surrounding the collapsing bubble, Fig. 15 shows the distributed pressures, determined for the maximum pressure wave heading towards the solid boundary, caused by an oscillating bubble for γ=1.0 and γ=2.0 during its first and second collapse. As seen, a shock wave was generated that reached the wall almost instantly, generating a sharp and localized pressure peak acting on the wall. After the shock wave was reflected, the pressure maximum shifted slightly (Fig. 15, top), and the reflected wave spread radially along the surface. When the bubble rebounded, toroidal collapse structures formed and caused the generation of secondary directional shock waves that reached the wall.

For γ=2.0, the bubble’s collapse was more spherical, due to its greater distance from the wall. Fig. 15 shows the pressure wave at the end of the bubble’s first and the second collapses. This pressure wave traveled a greater distance before impacting the wall. During this propagation, it spread and lost strength. As a result, the wall pressure was more evenly distributed and lasted for a longer time. Due to the bubble’s increased distance to the wall and its spherical shape, the wave energy spread more spherically. The level of condensation depended on its collapse intensity and, as its collapse was weaker, condensation was also less pronounced. Most of the rebound-induced waves decayed before reaching the wall. Although the secondary waves that were formed were weaker at the wall, they did not vanish entirely.

**Fig. 16:**
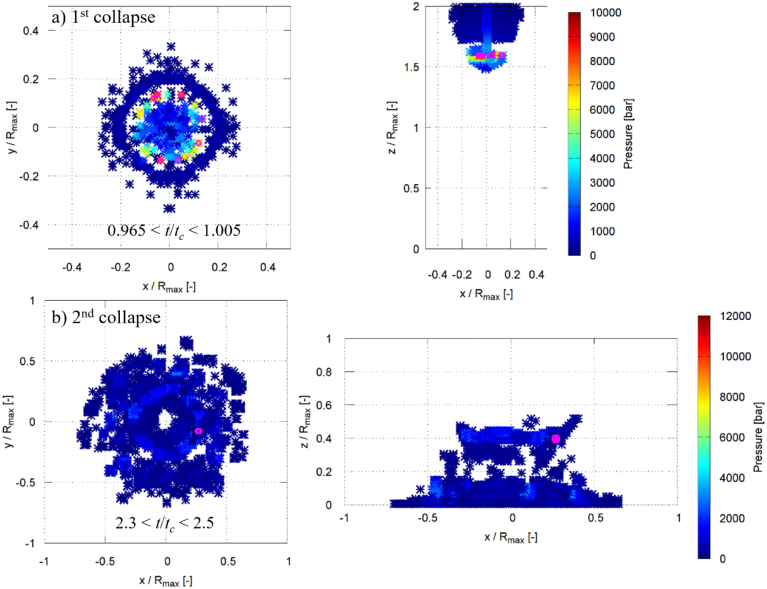
Spatial locations of the instantaneous maximum pressure inside the liquid phase for γ=2.0 during the (a) first collapse and (b) second collapse. The pressure maxima were identified within the non-dimensional time intervals indicated in each panel by magenta symbols.

**Fig. 17:**
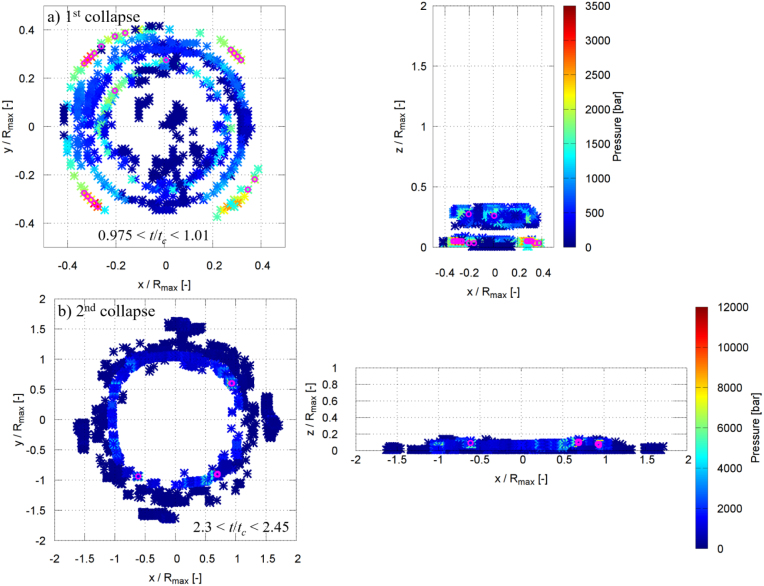
Spatial locations of the instantaneous maximum pressure inside the liquid phase for γ=1.0 during the (a) first collapse and (b) second collapse. The pressure maxima were identified within the non-dimensional time intervals indicated in each panel by magenta symbols.

Fig. 16, Fig. 17 show the spatial locations of the maximum pressure in the liquid phase for γ=2.0 and γ=1.0 during the first and second collapses, where the pressure maxima were identified within the non-dimensional time intervals indicated in the figures by magenta symbols. For γ=2.0, during the moments of the first collapse $\left(0.965\leq t/t_{c}\leq 1.005\right)$, several dominant pressure peaks are observed (Fig. 16a). These peaks are distributed at a radial distance of approximately $0.15\;R_{\max}$ and at a vertical position of about $1.6\;R_{\max}$, with pressure values ranging between 9000 and 9500 bar. Fig. 16b shows that during the second collapse $\left(2.3\leq t/t_{c}\leq 2.5\right)$, a single dominant pressure peak forms closer to the wall surface, at a radial distance of about $0.25\;R_{\max}$ and a vertical position of approximately $0.4\;R_{\max}$, reaching a pressure of roughly 10500 bar. The very high pressures are likely associated with shock-wave formation during bubble collapse. The regions with pressures exceeding 8000 bar are highly localized and typically confined to only one to a few computational control volumes on the fine grid, corresponding to a physical extent of approximately 20–60μm. Similar extreme local pressures (up to 2.4 GPa) have also been reported in molecular dynamics simulations of nanoscale bubble collapse [26], supporting the physical plausibility of such values. Although the nanoscale dynamics differ, these results still indicate that very high local pressures can occur during rapid collapse. A grid refinement study shows that finer discretization leads to higher peak pressures due to improved resolution of the shock wave. However, the present grid does not fully resolve the shock thickness; thus, the simulations capture the presence and propagation of shock waves but not their internal structure, and the peak pressures should be interpreted as resolution-dependent. The regions exceeding 8000 bar are highly localized and typically confined to single or few computational control volumes, rather than forming spatially extended regions.

For γ=1.0, the first collapse $\left(0.975\leq t/t_{c}\leq 1.01\right)$ is characterized by several pressure maxima mainly concentrated at two locations (Fig. 17a): one at a radial and vertical position of approximately ($0.25\;R_{\max}$, $0.25\;R_{\max}$), and another at ($0.4\;R_{\max}$, $0.1\;R_{\max}$). The corresponding pressure values lie between 2500 and 3000 bar. During the second collapse $\left(2.3\leq t/t_{c}\leq 2.45\right)$, several dominant pressure peaks are observed at a radial distance of about $1.0\;R_{\max}$ and a vertical position of approximately $0.1\;R_{\max}$, with pressures ranging from 8000 to 11100 bar (Fig. 17b). In all cases, the maximum pressure in the liquid exceeds the maximum pressure measured at the solid boundary. These localized liquid-phase pressure maxima act as the source of the pressure waves that impinge on the rigid plate, and their propagation and attenuation govern the maximum pressure ultimately experienced by the wall, as discussed in the following section.

**Fig. 18:**
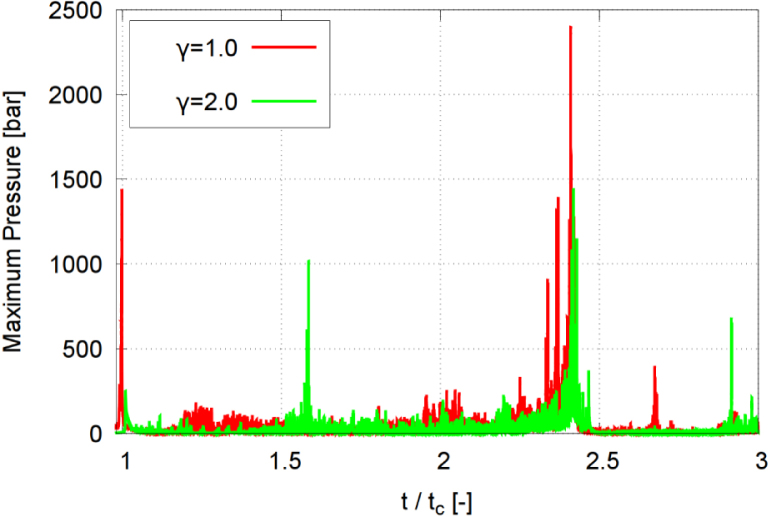
Maximum pressure acting on the wall at each time step, obtained with the stand-off distances γ=1.0 and γ=2.0, and time t normalized against collapse time $t_{c}$.

#### 6.1.3 Maximum pressure on the wall

Fig. 18 plots the time histories of computed maximum wall pressure showing the effect of the stand-off distance γ for the two cases γ=1.0 and γ=2.0. The reported pressure corresponds to the maximum pressure acting on the wall at each time step. As seen, the wall experienced a rapid and sharp pressure peak. For γ=2.0, the rise time of the pressure was larger. These differences resulted from the collapse strength, the phase change rate, and the particular spatial flow structure. For the greater stand-off distance the jet impact and the shock wave at the wall were less pronounced. Also, the combined effect of jet and shock loading was especially strong for the smaller γ, and this effect decreased markedly for a greater γ.

**Fig. 19:**
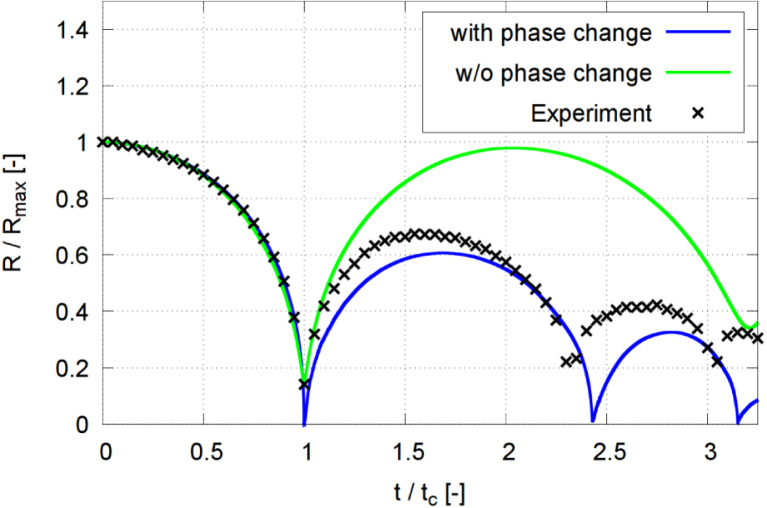
Normalized equivalent bubble radius $R/R_{\text{max}}$ in each time step, obtained from a simulation initialized at $R_{\text{max}}$, and another simulation performed without phase change (transition), for γ=2.0, and with time t normalized against collapse time $t_{c}$.

### 6.2 Effect of phase transition

To investigate the effect of phase change on bubble dynamics, we performed three numerical simulations with and without the phase transition model under identical conditions. Fig. 19 presents time histories of the evolution of the equivalent normalized bubble radius $R/R_{\text{max}}$ for a stand-off distance γ=2.0. The bubble dynamics was computed with (blue line) and without (green) phase transition. For the case without phase transition a small bubble filled with non-condensable gas was initialized at high pressure, so that its growth process was accounted for. Both simulations solved the energy equation. The experimental measurements (black crosses) served as a comparative reference for bubble dynamics.

When the phase-change model was excluded, our simulation predicted a relatively weak collapse, with the minimum dimensionless equivalent radius reaching 0.143. The bubble’s rebound size was much more pronounced (compared to the case with phase transition), attaining an $R/R_{\text{max}}=0.98$ at $t/t_{c}=2.03$. This behavior was consistent with earlier models without condensation [71], [72], where the absence of phase change maintained the high pressure inside the bubble. The elevated internal pressure cushioned the bubble’s collapse, reducing its intensity and leading to a stronger rebound than observed in the experiments.

The phase transition model fundamentally altered bubble dynamics. The minimum normalized equivalent radius decreased to $R/R_{\text{max}}=0.0025$, and the rebound normalized bubble radius reached $R/R_{\text{max}}=0.6273$ at $t/t_{c}=1.69$, in close agreement with the experiment. The minimum bubble size at its second collapse of $R/R_{\text{max}}=0.024$ showed that subsequent oscillations were strongly damped by condensation and evaporation.

**Fig. 20:**
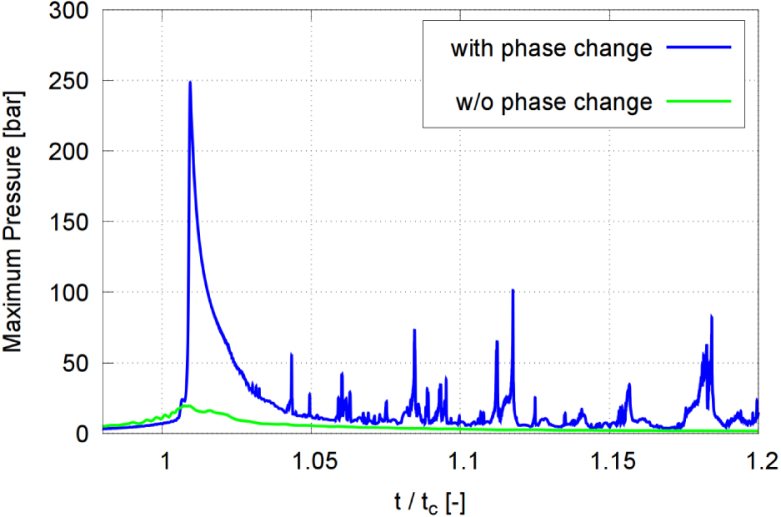
Maximum pressure in each time step, obtained with and without considering phase change (transition) for γ=2.0, and with time t normalized against collapse time $t_{c}$.

**Fig. 21:**
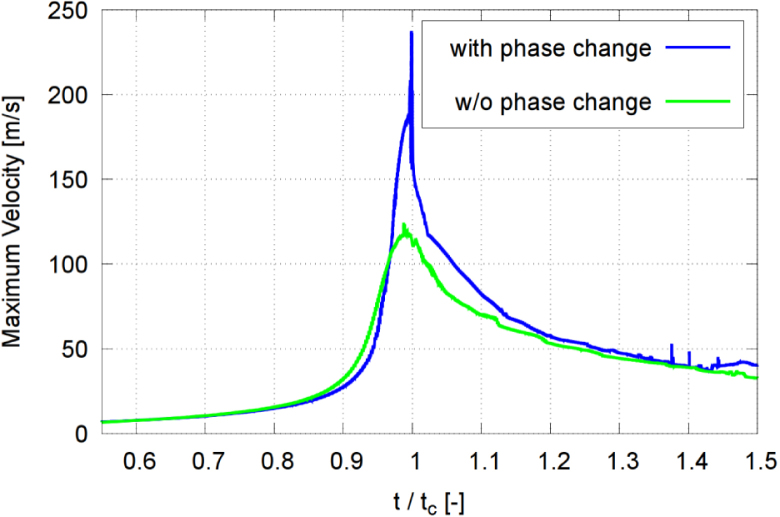
Maximum velocity in each time step, obtained with and without considering phase change (transition) for γ=2.0, and with time t normalized against collapse time $t_{c}$.

As seen in Fig. 20, the maximum pressure at the solid wall rose from 20 bar (without phase transition) (green line) to 250 bar (with phase transition) (blue line) and, as seen in Fig. 21, the maximum liquid velocity increased from 124 m/s (without phase transition) (green line) to 237 m/s (with phase transition) (blue line). Similar trends were reported by Nguyen et al. [72], who showed that, without phase transition, the predicted pressure peak is reduced to about 55% to 60% compared to the case with phase transition. The difference was caused by the intense condensation at collapse. Our simulations confirmed that neglecting phase transition led to an underestimated collapse intensity and an overestimated rebound, while including phase transition resulted in a more violent collapse, a stronger wall loading, and rebound characteristics that compared more favorably to experimental measurements.

#### 6.2.1 Modeling the growth phase of a laser-induced bubble

To model the growth phase of a laser-induced cavitation bubble, we performed a simulation starting with a computational domain entirely filled with water at rest. The temperature in the entire domain equaled the ambient temperature, and the pressure field corresponded to the hydrostatic pressure. For the initialization, a source term was introduced into the energy equation, corresponding to a laser energy of 20 mJ. The source term acted on a spherical region with a radius of 10μm. We used the ideal gas law, and obtained the vapor pressure as a function of temperature using the August–Roche–Magnus equation (Eq. (16)). When this pressure exceeded the local pressure of the plasma, a vapor bubble formed, and this vapor bubble continued to grow. Our modeling is questionable because the plasma cannot be modeled by a continuum approach. Likewise, the temperature-dependent vapor pressure was only defined up to the critical temperature $T_{crit}$. The maximum temperature in the plasma significantly exceeded this value in the initial time steps of the simulation. After about 3 ms, the maximum temperature fell below the critical value. Alternatively, bubble growth can be simulated by increasing initial pressure and suppressing phase transition during the bubble expansion phase, as demonstrated by Phan et al. [27]. Another strategy involves incorporating non-condensable gases alongside vapor in the initial phase. Our approach offers an alternative to these techniques. While each of these models has its limitations and conceptual challenges, they provide viable frameworks for simulating the growth phase.

**Fig. 22:**
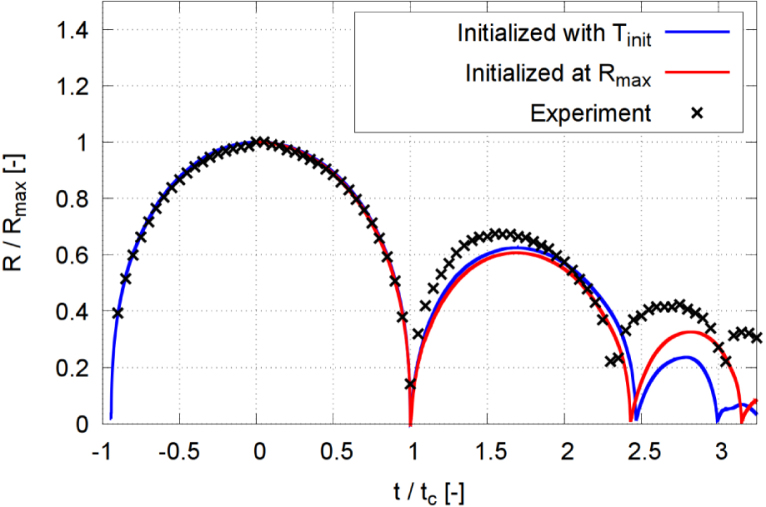
Normalized equivalent bubble radius $R/R_{\text{max}}$ in each time step, obtained from a simulation initialized at $R_{\text{max}}$, and another simulation initialized with high temperature considering the initial growing phase of the bubble for γ=2.0, and with time t normalized against collapse time $t_{c}$.

For γ=2.0, Fig. 22 shows that the temporal evolution of the normalized equivalent bubble radius (blue line) was similar to the corresponding evolution during bubble initialization at $R_{max}$ (red line). As seen, both curves compare favorably with the experimental measurement (black symbols). Despite the unrealistic modeling of the plasma during bubble initialization, the growth phase of the bubble’s first collapse cycle was also well captured and compared favorably with the experimental data.

### 6.3 Thermal effects

**Fig. 23:**
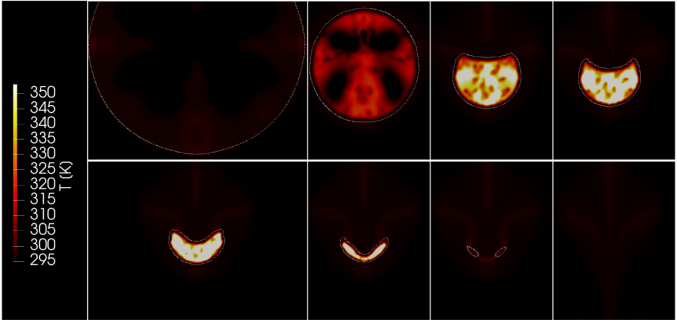
Temperature fields surrounding the bubble during its first collapse for γ=2.0, showing the fully grown bubble, the bubble during its collapse and jet formation, and the jet penetrating the bubble.

To understand the thermal impact of bubble collapse near a solid boundary, we also analyzed, in addition to the maximum pressure in the liquid and on the wall, the temperature distribution inside and around the bubble. To do so, we considered the stand-off distance of γ=2.0, where the solid boundary influenced bubble shape, jet formation, and collapse dynamics. Fig. 23 shows temperature contours at different stages of the bubble’s collapse and rebound. The attached color bar indicates the temperature, ranging from 290 to 350 K.

**Fig. 24:**
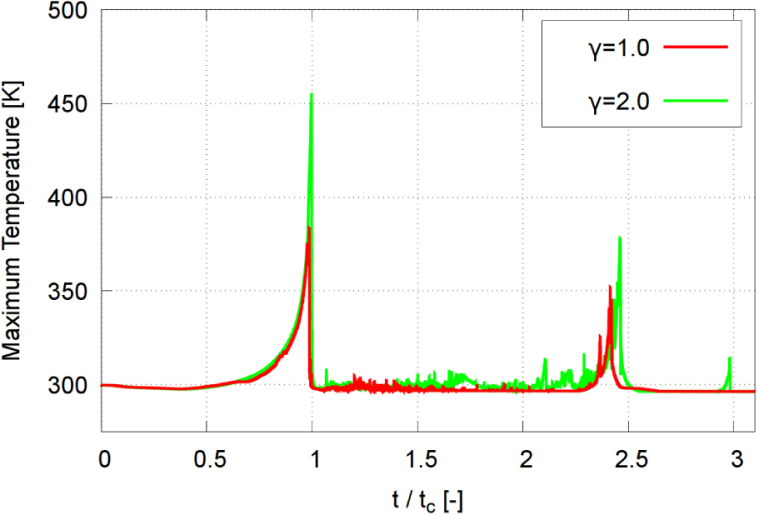
Maximum temperature inside the bubble in each time step, for γ=1.0 and γ=2.0, and with time t normalized against collapse time $t_{c}$.

At the end of the collapse, the gas inside the bubble was rapidly compressed by the motion of the surrounding liquid. This process generated strong heating inside the vapor phase, with the peak temperature rising to about 460 K near the center of the bubble, as indicated in Fig. 24, which presents the time history of the maximum temperature inside the bubble during its first collapse for the two stand-off distances γ=1.0 (red line) and γ=2.0 (green line).

This heating was caused by the (nearly adiabatic) compression as the collapse occurred quickly and did not allow significant heat exchange between the vapor and the surrounding liquid. The temperature was highest just before the bubble reached its minimum volume. The temperature inside the bubble was not uniformly distributed. In a spherically symmetric collapse, the vapor temperature could have increased beyond 1000 K. However, the presence of the solid boundary reduced the strength of the compression and caused the maximum temperature inside the bubble to decrease [73].

As the rebound began, the bubble expanded and vaporization occurred. This phase transition required energy in the form of latent heat, which was extracted from the surrounding liquid. As a result, the bubble surface, especially near the wall, cooled during this stage.

Experimental measurement of temperatures induced by bubble collapse—whether within the fluid phase or at the solid boundary remains exceptionally challenging. To the authors’ knowledge, no experimental data of this nature has been reported to date. However, molecular dynamics based simulations of nano bubble dynamics conducted by Rezaee et al. [26] demonstrate temperature profiles that align with the trends observed in our study.

**Fig. 25:**
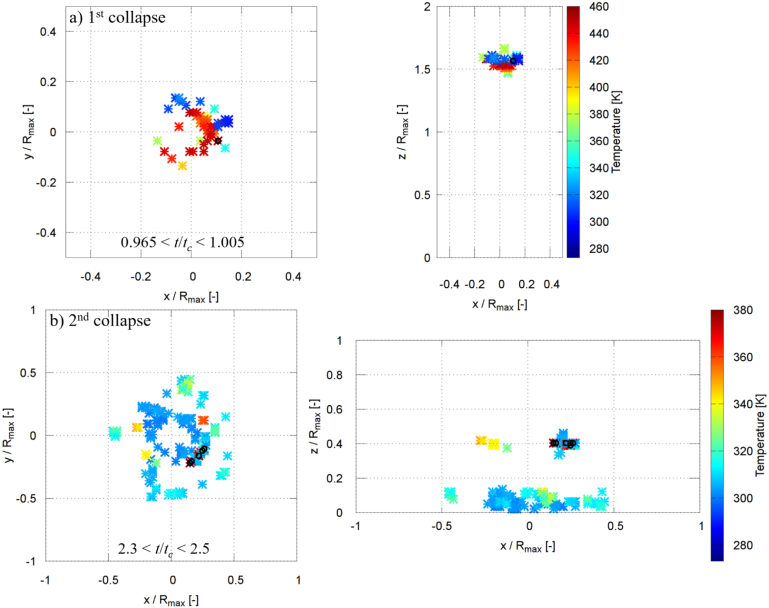
Spatial locations of the instantaneous maximum temperature inside the fluid for γ=2.0 during the (a) first collapse and (b) second collapse. The temperature maxima were identified within the non-dimensional time intervals indicated in each panel by black symbols.

**Fig. 26:**
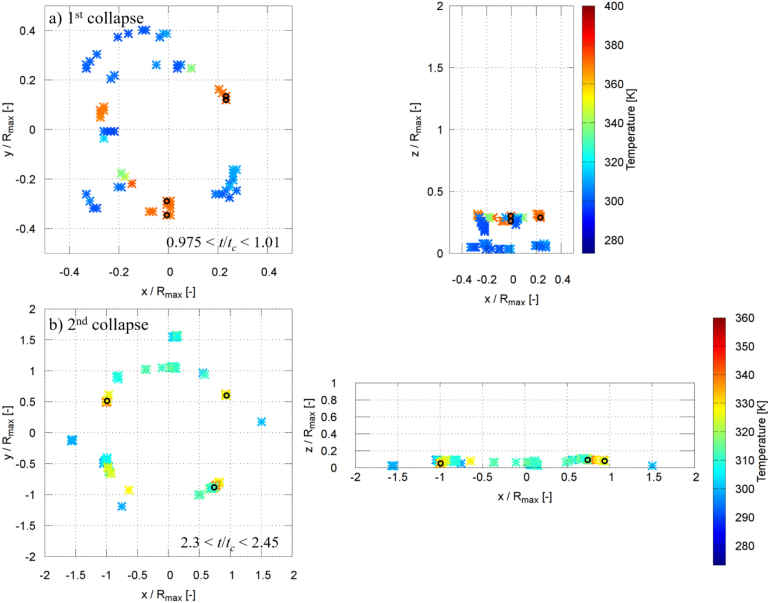
Spatial locations of the instantaneous maximum temperature inside the fluid for γ=1.0 during the (a) first collapse and (b) second collapse. The temperature maxima were identified within the non-dimensional time intervals indicated in each panel by black symbols.

Fig. 25, Fig. 26 show the spatial locations of the maximum temperature, which occurred in the vapor phase, for γ=2.0 and γ=1.0 during the first and second collapses, where the temperature maxima were identified within the non-dimensional time intervals indicated in the figures. In both cases, the dominant temperature peaks appear at locations closely aligned with the corresponding pressure maxima (Fig. 16, Fig. 17), indicating strong coupling between vapor compression and liquid-phase pressure generation. However, unlike pressure, the highest temperatures are confined to the vapor region. For γ=2.0, during the first collapse $\left(0.965\leq t/t_{c}\leq 1.005\right)$, the peak vapor temperature reaches values between 440 and 456 K (Fig. 25a). During the second collapse $\left(2.3\leq t/t_{c}\leq 2.5\right)$, the maximum vapor temperature is lower, ranging between 360 and 378 K, despite the higher liquid-phase pressure levels observed at this stage (Fig. 25b). For γ=1.0, the temperature rise is generally weaker: during the first collapse $\left(0.975\leq t/t_{c}\leq 1.01\right)$, the peak vapor temperature lies between 370 and 383 K (Fig. 26a), while during the second collapse $\left(2.3\leq t/t_{c}\leq 2.45\right)$ it further decreases to values between 340 and 351 K (Fig. 26b). A similar dependence of the maximum temperature on γ was reported by Yang et al. [74], who showed that increasing γ leads to higher peak temperatures during bubble collapse. In their results, the maximum temperature for γ=2.0 was approximately 15% higher than that for γ=1.0, which is comparable to the trend observed here. These results highlight a clear separation between the locations of maximum pressure and maximum temperature, with pressure peaking in the surrounding liquid and temperature peaking within the collapsing vapor. Notably, the maximum pressure is consistently generated in the liquid phase, whereas the maximum temperature is confined to the vapor phase during bubble collapse.

It is important to note that while pressures exceed 10,000 bar, the maximum vapor temperature reaches only 460 K. This apparent discrepancy arises because pressure and temperature peaks occur in different phases: extreme pressures are generated in the liquid by shock-wave compression, whereas the temperature peak occurs in the vapor, where pressures remain below 10 bar. Due to rapid phase transition and the absence of non-condensable gas, vapor condenses once local pressure exceeds saturation pressure, limiting adiabatic heating. Consequently, liquid compressibility governs the pressure peaks, while vapor temperatures remain moderate, consistent with molecular dynamics results [26]. Although not directly comparable due to the nanoscale nature of those simulations, they similarly indicate moderate temperature increases despite very high local pressures.

### 6.4 Effect of salinity

Adding solute like NaCl to distilled water changes its physical properties, such as density, viscosity, surface tension, and vapor pressure. The salinity of seawater is about 35 g/kg. At 20 °C, the density of seawater is about 1025 kg/m${}^{3}$ compared to the density of 998 kg/m${}^{3}$ for pure water [75]. The dynamic viscosity of seawater is about 8 to 10% higher than that of pure water [76], and the surface tension of seawater, being 0.073 N/m, i. e., slightly higher than that of fresh water, being 0.074 N/m [76]. As the vapor pressure decreases slightly with salinity [75], it reduces the driving force for bubble growth. Higher density increases the inertia of the surrounding liquid, while higher viscosity increases energy dissipation during bubble dynamics. These effects lead to smaller bubble sizes and less violent collapses. In macroscopic cavitation, the higher surface tension raises the cavitation inception threshold, reducing the number of bubbles that form, however, it improves the stability. Saltwater also tends to have fewer active nuclei and possibly smaller critical radius [76], which further reduces the number of bubbles that can grow. In addition, gas solubility decreases by about 27% [76].

We experimentally investigated the effect of salinity on the collapse dynamics of a laser-induced single vapor bubble. To isolate the influence of dissolved salts, three NaCl concentrations were prepared: distilled water, seawater of 35 g/kg salinity, and a saline solution with a high concentration of 100 g/kg. For each salinity case, bubble dynamics were examined for three stand-off distances: in a free field, for a distance of 3.0 mm, and for a distance of 0.6 mm from a solid boundary. The 3.0 mm distance corresponded to γ=2.0; the 0.6 mm distance, to γ=0.4.

**Fig. 27:**
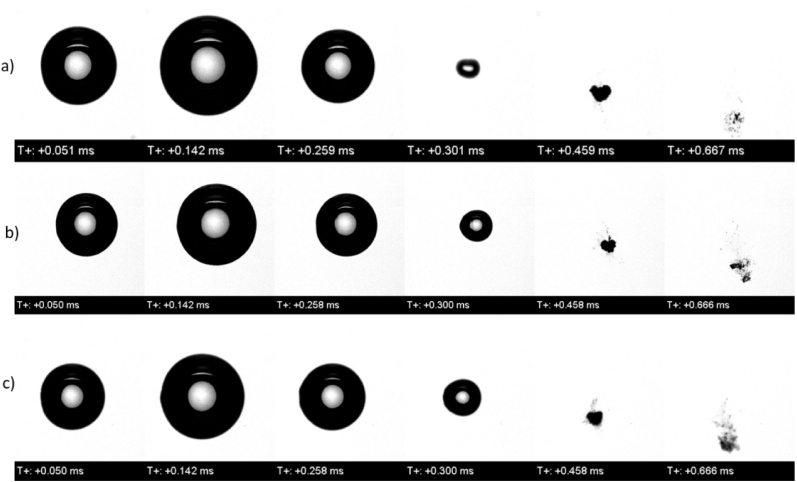
For the bubble oscillating in the free field, experimentally measured time sequences of the bubble oscillating in a solution of three differing salinity levels: distilled water of 0 salinity (sequence a), seawater of 35 g/kg salinity (sequence b), and a solution with a highly concentrated salinity of 100 g/kg (sequence c).

**Fig. 28:**
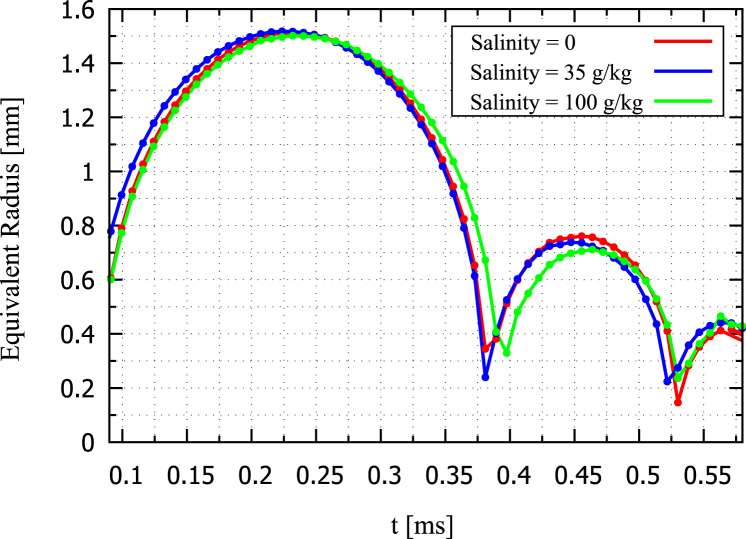
Experimentally measured time histories of the normalized equivalent bubble radius $R/R_{\text{max}}$ in free field for different salinity levels.

For the free field case, Fig. 27 presents three experimentally measured time sequences of the bubble oscillating in a solution of three differing salinity levels: distilled water of 0 salinity (sequence a), seawater of 35 g/kg salinity (sequence b), and a solution with a highly concentrated salinity of 100 g/kg (sequence c). The first three frames represent the bubble’s primary oscillation cycle. This showed that the maximum bubble size remained mostly unchanged across different salinities, which was due to the fixed laser energy input. Fig. 28 presents the corresponding experimentally measured time histories of the normalized equivalent bubble radius $R/R_{\text{max}}$ for the bubble oscillating in these three solutions of 0 salinity (red line), 35 g/kg (blue line), and 100 g/kg (green line). As seen, a slight decrease in maximum radius with increasing salinity was still observed, indicating the subtle influence of fluid properties like vapor pressure and density.

More noticeable differences appeared during the bubble’s rebound. Higher salinity resulted in a visibly smaller rebounded equivalent bubble radius, indicating increased energy dissipation or an altered phase transition. As the bubble rebounded and gradually generated mist and vapor structures after its sequence a, it became more compact with increasing salinity, which was likely due to higher density and surface tension that stabilized the vapor remnants.

**Fig. 29:**
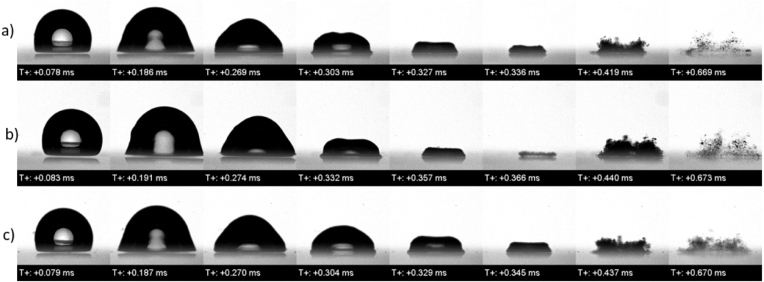
For the 0.6 mm stand-off distances, i. e. for γ=0.4, experimentally measured time sequences of the bubble oscillating in a solution of three differing salinity levels: distilled water of 0 salinity (sequence a), seawater of 35 g/kg salinity (sequence b), and a solution with a highly concentrated salinity of 100 g/kg (sequence c).

For the 0.6 mm stand-off distances, i. e., for γ=0.4, Fig. 29 also presents an experimentally measured time sequence of the bubble oscillating in a solution of three differing salinity levels: distilled water of 0 salinity (sequence a), seawater of 35 g/kg salinity (sequence b), and a solution with a highly concentrated salinity of 100 g/kg (sequence c). The image sequence of this case showed that the overall dynamics were similar across all salinities, the jet was slower, and the rebound was weaker at higher salinity. Mist and vapor residue were minimal immediately after collapse, suggesting that the jet expelled vapor from the field of view. However, after the second cycle, more structured mist was observed in the high salinity case, while vapor was more scattered at low salinity.

**Fig. 30:**
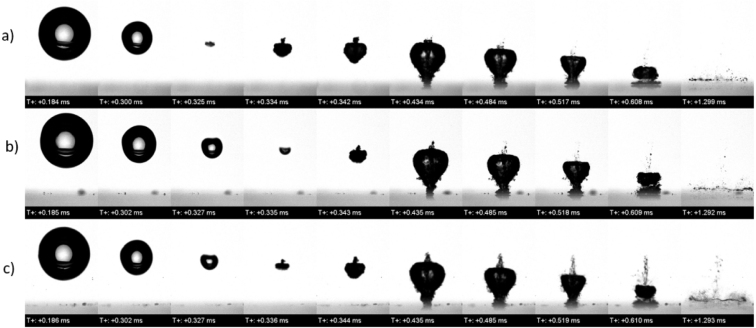
For the 3.0 mm stand-off distances, i. e. for γ=2.0, experimentally measured time sequences of the bubble oscillating in a solution of three differing salinity levels: distilled water of 0 salinity (sequence a), seawater of 35 g/kg salinity (sequence b), and a solution with a highly concentrated salinity of 100 g/kg (sequence c).

For the 3.0 mm stand-off distances, i. e., for γ=2.0, Fig. 30 also presents an experimentally measured time sequence of the bubble oscillating in a solution of three differing salinity levels: distilled water of 0 salinity (sequence a), seawater of 35 g/kg salinity (sequence b), and a solution with a highly concentrated salinity of 100 g/kg (sequence c). Here, the collapse remained mostly symmetric and, at the end of cycle a, deformation caused by the jet was observable at t=0.325ms. In the high salinity case, the rebound was weaker, and vapor was still visible at t=0.485ms. Although these features were also observed in the lower and no salinity cases, they disappeared gradually. However, in the 100 g/kg case, vapor remained visible until t=1.293ms, and the bubble was noticeably smaller. Fine mist lines were still present, in contrast to the lower salinity cases, where vapor already condensed or dispersed.

These results showed that increasing salinity modified the fluid properties density, viscosity, and surface tension, leading to stronger damping and more persistent vapor structures. Higher salinity reduced bubble rebound and promoted the formation of structured mist. These remnants could have served as nucleation sites for subsequent cavitation activity. The strongest and fastest collapsed occurred at low salinity and small stand-off distances, where inertia was dominant. At higher salinity and longer distances, the bubble dynamics became more diffusive, and the residual vapor was more structured and longer-lasting. Thus, although high salinity appeared to reduce bubble size, it may have enhanced cavitation persistence with stable vapor remnants.

## 7 Conclusions

We numerically investigated the dynamics of a single vapor bubble collapsing near a solid boundary, considering compressibility, phase transition, and thermodynamic effects in a three-dimensional domain. Our respective numerical simulations solved the conservation equations of mass, momentum, and energy together with a transport equation for the liquid volume fraction to consider mass transfer. We verified our numerical method by conducting a systematic discretization study. Validation against the experiments of a laser-induced bubble showed that the phase transition had major effects on bubble dynamics and the associated pressures, velocities, and temperatures in the flow field surrounding the bubble after its first collapse.

Accounting for phase transition yielded stronger collapses, higher wall pressures, and more pronounced rebounds that agreed closely to experimental measurements. Neglecting phase transition underestimated the bubble’s collapse intensity and overestimated its rebound. Our results showed that condensation during collapse reduced vapor volume and lowered internal pressure, while evaporation affected the bubble’s rebound. Thermodynamic effects appeared mainly as adiabatic heating during a collapse, reaching about 460 K for a stand-off distance of γ=2.0. Our three-dimensional simulations led to asymmetric jets and toroidal collapse structures, and our sensitivity study illustrated the effect of condensation and evaporation rates on bubble dynamics and the associated flow.

We studied salinity effects experimentally. Increasing salinity changed the liquid properties and led to weaker bubble rebounds and more persistent vapor remnants. These vapor remnants, when acting as nucleation sites, may have influenced the persistence of cavitation. The strongest and fastest collapses were observed at low salinity levels and small stand-off distances, while higher salinity levels produced diffusive and long-lasting vapor structures.

## CRediT authorship contribution statement

**Udo Lantermann:** Writing – original draft, Validation, Methodology, Investigation, Conceptualization. **Gohar Moloudi:** Writing – original draft, Visualization, Investigation, Formal analysis. **Mazyar Dawoodian:** Writing – original draft, Visualization, Investigation, Formal analysis, Conceptualization. **Hemant J. Sagar:** Visualization, Methodology, Investigation. **Ould el Moctar:** Writing – review & editing, Writing – original draft, Supervision, Resources, Project administration, Methodology, Conceptualization.

## Funding

The research was funded by the 10.13039/100000006Office of Naval Research GRANT14309963 TR: Dr. Richard Meyer and by the German Research Foundation (DFG) under the project grant 533951202. We gratefully acknowledge the computing time granted by the Center for Computational Sciences and Simulation (CCSS) of the University of Duisburg–Essen provided on the supercomputer amplitUDE (DFG grant INST 20867/423-1 FUGG) at the Zentrum für Informations- und Mediendienste (ZIM).

## Declaration of competing interest

The authors declare that they have no known competing financial interests or personal relationships that could have appeared to influence the work reported in this paper.
